# Dissection of genetic architecture for glucosinolate accumulations in leaves and seeds of *Brassica napus* by genome‐wide association study

**DOI:** 10.1111/pbi.13314

**Published:** 2019-12-25

**Authors:** Sheng Liu, Huibin Huang, Xinqi Yi, Yuanyuan Zhang, Qingyong Yang, Chunyu Zhang, Chuchuan Fan, Yongming Zhou

**Affiliations:** ^1^ National Key Laboratory of Crop Genetic Improvement Huazhong Agricultural University Wuhan China; ^2^ Key Laboratory of Biology and Genetic Improvement of Oil Crops Ministry of Agriculture and Rural Affairs Oil Crops Research Institute of the Chinese Academy of Agricultural Sciences Wuhan China; ^3^ Hubei Key Laboratory of Agricultural Bioinformatics College of Informatics Huazhong Agricultural University Wuhan China

**Keywords:** rapeseed (*Brassica napus*), metabolism, glucosinolates, GWAS, *MYB28*

## Abstract

Glucosinolates (GSLs), whose degradation products have been shown to be increasingly important for human health and plant defence, compose important secondary metabolites found in the order Brassicales. It is highly desired to enhance pest and disease resistance by increasing the leaf GSL content while keeping the content low in seeds of *Brassica napus*, one of the most important oil crops worldwide. Little is known about the regulation of GSL accumulation in the leaves. We quantified the levels of 9 different GSLs and 15 related traits in the leaves of 366 accessions and found that the seed and leaf GSL content were highly correlated (*r* = 0.79). A total of 78 loci were associated with GSL traits, and five common and eleven tissue‐specific associated loci were related to total leaf and seed GSL content. Thirty‐six candidate genes were inferred to be involved in GSL biosynthesis. The candidate gene *BnaA03g40190D* (*BnaA3.MYB28*) was validated by DNA polymorphisms and gene expression analysis. This gene was responsible for high leaf/low seed GSL content and could explain 30.62% of the total leaf GSL variation in the low seed GSL panel and was not fixed during double‐low rapeseed breeding. Our results provide new insights into the genetic basis of GSL variation in leaves and seeds and may facilitate the metabolic engineering of GSLs and the breeding of high leaf/low seed GSL content in *B. napus*.

## Introduction

Glucosinolates (GSLs) are sulphur‐ and nitrogen‐containing secondary metabolites, some of which are well known for their anticarcinogenic properties in humans and their antiherbivore and antimicrobial properties in plants (Baskar *et al.*, 2012; Bradbury *et al.*, 2007, p. 14; Soundararajan and Kim, 2018). GSLs are commonly found in the order Brassicales, which include economically and nutritionally important *Brassica* crop and vegetable species, such as rapeseed (*Brassica napus*), cabbage (*Brassica oleracea*) in addition to the model plant *Arabidopsis thaliana* (Halkier and Gershenzon, 2006). *B. napus* is grown primarily as a source of edible oil and for its protein‐rich seedcake for animal feed. A high content of GSLs, mainly 2‐hydroxy‐3‐butenyl GSL, in the seedcake can cause goitres and has other harmful effects on animal nutrition (Griffiths *et al.*, 1998). Breeders have dramatically reduced the level of seed GSL from> 100 µmol/g to < 30 µmol/g through the introgression of alleles from the Polish cultivar ‘Bronowski’ (Kondra and Stefansson, 1970). However, this reduction tends to be associated with a concomitant reduction of GSL content in the leaves, causing cultivars to be more susceptible to pests, birds and pathogens (Mithen, 1992). For this reason, it is desirable to enhance the protective effects of rapeseed by manipulating the leaf profile without reducing seed quality. Therefore, it is necessary to better understand the genetic basis of GSL biosynthesis and accumulation in the leaves and seeds of rapeseed.

Glucosinolates are derived from amino acids and thus can be classified into three groups according to their amino acid precursor: aliphatic GSLs, derived from amino acids of Ala, Leu, Ile, Val, and Met; benzenic GSLs, derived from Phe or Tyr; and indolic GSLs, derived from Trp (Halkier and Gershenzon, 2006). These three groups of GSLs are independently biosynthesized and regulated by different sets of genes (Kliebenstein *et al.*, 2001a). GSL biosynthesis is a tripartite pathway that includes three stages: side chain elongation of amino acids, core structure formation and secondary side chain modification. The pathway has been best characterized in *A. thaliana*, in which nearly all genes involved in the three biosynthesis stages have been identified (Sønderby *et al.*, 2010, Figure 1). GSLs are synthesized mainly in source tissues such as leaves and silique walls and then transported to embryos through phloem by specific transporters (Chen *et al.*, 2001; Ellerbrock *et al.*, 2007; Nour‐Eldin and Halkier, 2009). Two transporters, GTR1 and GTR2, have been reported in *A. thaliana*. The *gtr1 gtr2* double mutant did not accumulate GSLs in its seeds, but GSLs over‐accumulated more than tenfold in the leaves and silique walls (Nour‐Eldin *et al.*, 2012).

**Figure 1:**
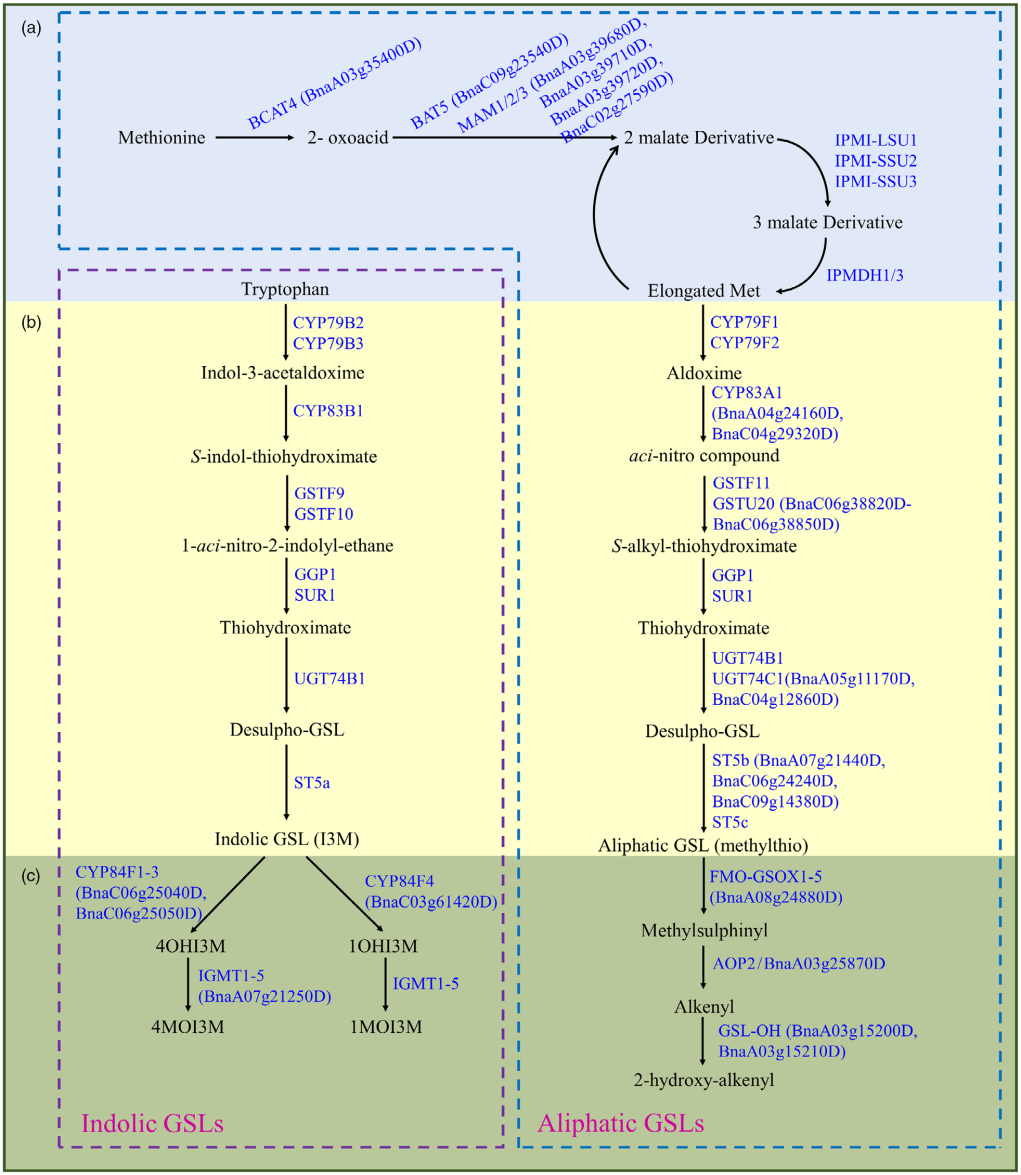
The aliphatic and indolic GSL biosynthesis pathways in Brassicaceae. (a) Side chain elongation. (b) Biosynthesis of the core GSL structure. (c) Secondary side chain modification. The candidate GSL genes identified in this study are listed in brackets. The figure was constructed according to the data from several publications (Pfalz et al., 2011; Sønderby et al., 2010).

Over the last few decades, bi‐parental mapping populations have been extremely valuable for the detection of quantitative trait loci (QTLs) responsible for quantitative variation in GSL profiles. Side chain elongation and hydroxylation in seeds or leaves are both controlled by two loci in populations derived from crosses between oilseed rape cultivars and synthetic *B. napus* lines (Magrath *et al.*, 1994; Parkin *et al.*, 1994). Three to five major QTLs control the total seed GSL content (Seed‐GSL) in several bi‐parental linkage populations derived from crosses between low‐ and high‐GSL accession, and 43 QTLs control this trait in a low‐GSL genetic background in *B. napus* (Fu *et al.*, 2015; Howell *et al.*, 2003; Toroser *et al.*, 1995; Uzunova *et al.*, 1995; Zhao and Meng, 2003). Feng *et al. *(2012) identified 105 metabolite QTLs for GSL compounds, among which 71 and 17 were seed and leaf specific, respectively. Compared with bi‐parental linkage mapping, genome‐wide association study (GWAS) offers more cost‐effective features for QTL localization as well as higher‐resolution for candidate gene identification and has recently been applied to rapeseed (Harper *et al.*, 2012; Körber *et al.*, 2016; Li *et al.*, 2014; Lu *et al.*, 2014; Qu *et al.*, 2015; Wang *et al.*, 2018). Using associative transcriptomics, Harper *et al. *(2012) discovered in a population of 53 *B. napus* lines that three loci on chromosomes A9, C2 and C9 were significantly associated with seed GSL content. Furthermore, deletions of orthologs gene of the *AtMYB28* (which controls aliphatic GSL biosynthesis) on chromosomes A9 and C2 were considered to lead to low seed GSL content. Lu *et al. *(2014) increased the population to 101 *B. napus* lines and inferred that 26 genes, including *BnaA.GTR2a* and *BnaC.HAG3b* (orthologs gene of the *AtMYB28*), were associated with seed GSL content. Using 24 256 SNPs from a *Brassica* 60K SNP array and a panel of 472 rapeseed accessions, Li *et al. *(2014) suggested that different copies of *MYB28* on chromosomes A9, C2, C7 and C9 were responsible for the seed GSL content. Using high‐throughput genome resequencing, Wang *et al. *(2018) identified 49 loci associated with seed GSL content and 27 candidate genes involved in GSL biosynthesis and breakdown. Previous studies examining GSL QTLs have mainly focused on the accumulation of seed GSLs; thus, efforts are needed to identify the factors controlling GSL quantitative variation in vegetative tissues and the key genes participating in the individual steps of the GSL biosynthesis pathway.

To better understand the genetic control of GSL in the leaves of *B. napus*, we analysed GSL metabolites in a panel of 366 accessions that were genotyped with a *Brassica* 60K SNP array and performed a GWAS to investigate and compare the genetic control of GSL accumulation in the leaves and seeds. We identified associated loci and candidate genes involved in aliphatic and indolic GSL biosynthesis pathways, including both common and specific loci associated with GSL accumulation in the two different tissues. We analysed the selection effect during the breeding of low‐GSL rapeseed cultivars and found that the *BnaA03g40190D* (*BnaA3.MYB28*) gene was not fixed and was responsible for high leaf/low seed GSL content. Our results provide new insights into GSL biosynthesis and may facilitate genetic manipulation and metabolic engineering of GSLs in *B. napus*.

## Results

### Aliphatic GSLs are the major type for GSL variation in leaves of *B. napus*

The GSL content in the leaves of 366 *B. napus* accessions, among which there were 168 with low total GSL in seeds, was measured at 90 days after sowing in two consecutive years (2013–2014 and 2014–2015). Six aliphatic GSL compounds and three indolic GSL compounds were detected via high‐performance liquid chromatography (HPLC), and an additional 15 descriptive variables from these measurements were defined for a total of 24 GSL traits in leaves (abbreviations and descriptions of GSL traits are listed in Table S1). These GSL phenotypic data exhibited continuous and wide variations, but did not fit a normal distribution (*P* < 0.001, Table 1, Figure S1–S4). The coefficient of variation (CV) of 24 GSL traits ranged from 0.17 (4C/TALI) to 2.08 (4MSO), and the CV of the aliphatic GSLs was higher than that of the indolic GSLs (Table 1). These results indicated that the GSL phenotypic variation in the leaves was caused mainly by aliphatic GSLs. As expected, the nine GSL compounds tended to be strongly correlated within the same type of GSL and less correlated between the aliphatic and indolic GSLs (Table S2). The strongest Spearman’s correlation among the nine GSL compounds occurred between 4OHB and 5PTEY, 4OHB and 5OHP (*r* = 0.89, *P* < 0.01), and the lowest correlations occurred between 4MSO and 1MOI3M (*r *= −0.03, *P *> 0.05, Table S2).

**Table 1:** Summary statistics of the 25 glucosinolate (GSL) traits

Trait	BLUPs				*r* †	*H* ^2^ (%)
	Mean ± SD (µmol/g)	Range (µmol/g)	CV	W		
Leaf‐GSL	1.84 ± 1.18	0.34–6.90	0.64	0.89***	0.75***	85.1
Seed‐GSL	49.92 ± 31.87	17.45–131.80	0.64	0.79***	0.90***	97.1
4OHB	0.38 ± 0.33	0.05–1.51	0.87	0.85***	0.78***	74.8
4MSO	0.02 ± 0.04	0–0.56	2.08	0.35***	0.59***	78.9
4BTEY	0.22 ± 0.20	0.02–1.94	0.89	0.75***	0.76***	82.8
5OHP	0.08 ± 0.10	0–0.81	1.29	0.69***	0.86***	93.0
5MSO	0.19 ± 0.16	0.02–1.28	0.84	0.81***	0.72***	76.7
5PTEY	0.56 ± 0.52	0.04–3.21	0.94	0.83***	0.80***	88.6
I3M	0.29 ± 0.10	0.13–0.69	0.34	0.91***	0.45***	64.0
4MOI3M	0.04 ± 0.01	0.02–0.09	0.26	0.96***	0.43***	53.2
1MOI3M	0.03 ± 0.01	0–0.10	0.47	0.81***	–	50.9
TALI	1.45 ± 1.16	0.12–6.15	0.80	0.89***	0.80***	85.4
TIND	0.35 ± 0.10	0.18–0.79	0.29	0.92***	0.45***	64.4
4C	0.62 ± 0.48	0.08–2.41	0.78	0.88***	0.77***	77.1
5C	0.83 ± 0.71	0.05–4.16	0.85	0.87***	0.81***	89.0
MSO	0.21 ± 0.18	0.02–1.31	0.84	0.80***	0.71***	77.7
OHAlk	0.46 ± 0.42	0.04–2.30	0.91	0.85***	0.81***	80.7
Alkenyl	0.78 ± 0.68	0.06–4.99	0.87	0.85***	0.79***	87.5
4MSO/4C	0.07 ± 0.12	0.01–0.83	1.70	0.51***	0.51***	84.4
5MSO/5C	0.33 ± 0.20	0.06–0.92	0.61	0.88***	0.75***	88.3
4C/TALI	0.40 ± 0.07	0.22–0.72	0.17	0.90***	0.39***	64.8
OHAlk/TALI	0.27 ± 0.09	0.05–0.64	0.35	0.98***	0.64***	78.0
Alkenyl/TALI	0.50 ± 0.15	0.07–0.81	0.31	0.95***	0.70***	82.2
4MO/TIND	0.14 ± 0.04	0.08–0.41	0.27	0.89***	0.39***	55.1
1MO/TIND	0.08 ± 0.03	0–0.27	0.45	0.89***	–	67.2

CV, coefficient of variation; SD, standard deviation; W, Shapiro–Wilk Statistics.

Abbreviations and descriptions of traits are explained in Table S1.

*** *P* < 0.001.

^†^ *r*, Spearman’s correlation coefficients of traits between two environments.

Analysis of variance (ANOVA) revealed that the genotype (G), environment (E) and genotype × environment interaction (G × E) have significant effects on the 24 GSL traits (*P* < 0.01, Table S3). Among the traits, the broad‐sense heritability was greatest (93.0%) for 5OHP, while the lowest (50.9%) was observed for 1MOI3M (Table 1). Significant correlations (0.39 ≤ *r* ≤ 0.86, *P* < 0.001) were observed between the two environments (Table 1). The results thus indicated that variation in GSL traits in rapeseed leaves was influenced largely by genetic effects and could be used for further genetic analyses.

### GSL accumulation in leaves and seeds is partially overlapped

**Figure 2:**
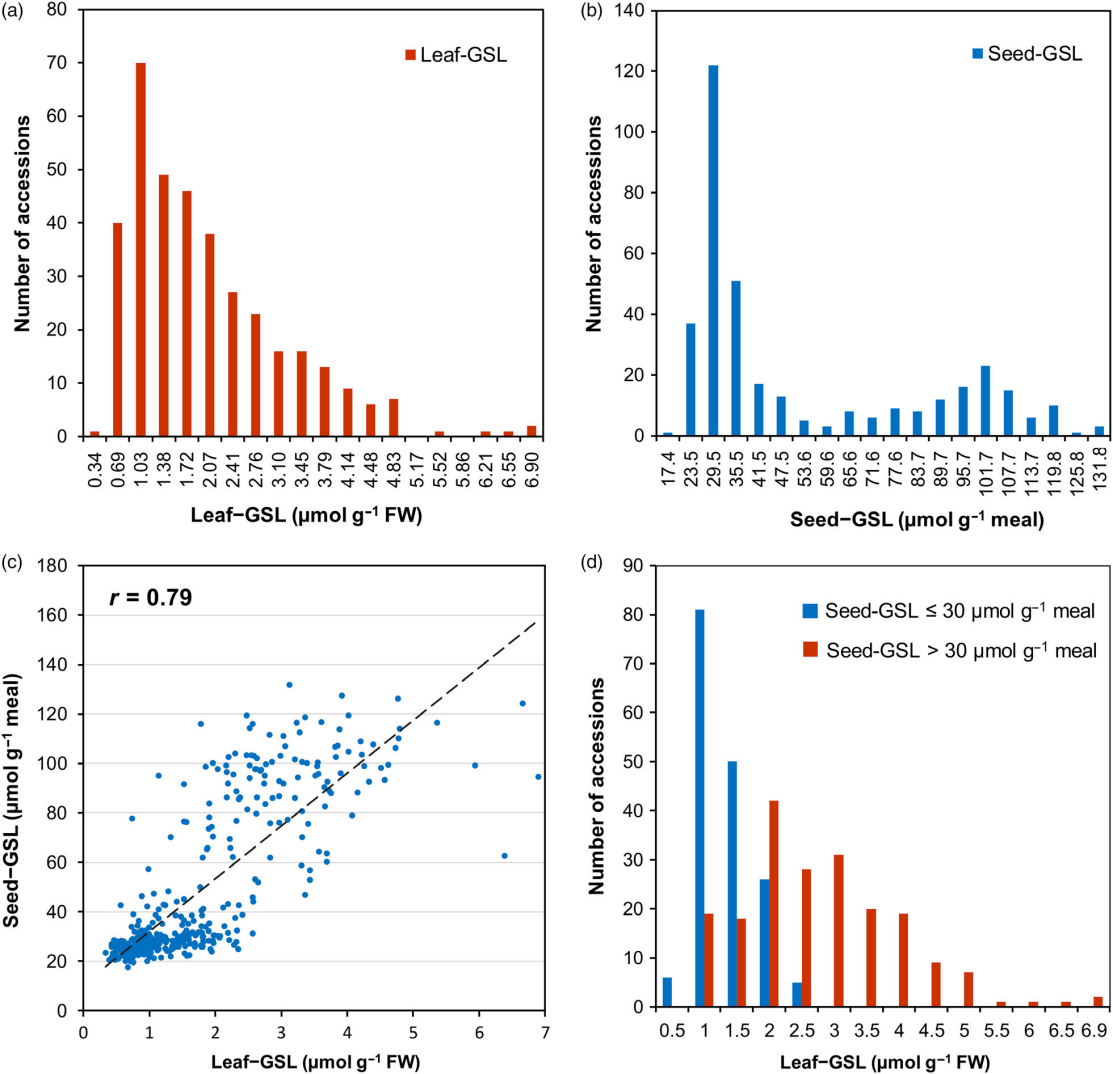
Variation in and correlations between Leaf‐GSL and Seed‐GSL. Distribution of Leaf‐GSL (a) and Seed‐GSL (b); (c) Correlation between Leaf‐GSL and Seed‐GSL. *r* represents the Spearman’s correlation coefficient; (d) Distribution of Leaf‐GSL when the Seed‐GSL was high (>30 µmol/g meal) and low (≤30 µmol/g meal).

Furthermore, the correlations between the total leaf and seed GSL content were analysed among the 366 *B. napus* accessions. The total leaf GSL content (Leaf‐GSL) ranged from 0.34 to 6.90 µmol/g fresh weight (FW), with an average of 1.84 ± 1.18 µmol/g FW, and the Seed‐GSL ranged from 17.45 to 131.80 µmol/g meal, with an average of 49.92 ± 31.87 µmol/g meal (Figure 2a, b, Table 1). These two traits were strongly correlated with a Spearman’s correlation coefficient of 0.79 (*P* < 0.01, Figure 2c). However, such a correlation did not mean that accessions with a relatively low Seed‐GSL always had a low Leaf‐GSL. A closer look at the variations found that there are genotypes with relatively higher GSLs within the subpopulations with low Seed‐GSL. For example, among the accessions with the Seed‐GSL low (≤30 µmol/g meal), their Leaf‐GSL ranged from 0.34 to 2.35 µmol/g FW; among the ones with the Seed‐GSL high (>30 µmol/g meal), their Leaf‐GSL ranged from 0.57 to 6.90 µmol/g FW (Figure 2d). These results suggested that GSL biosynthesis in the leaves and seeds was not completely independent, indicating that it is possible to select for higher levels of leaf GSLs in conjunction with low seed GSLs (high leaf/low seed GSL content).

### Candidate genes involved in GSL accumulations in leaves were identified by GWAS

**Figure 3:**
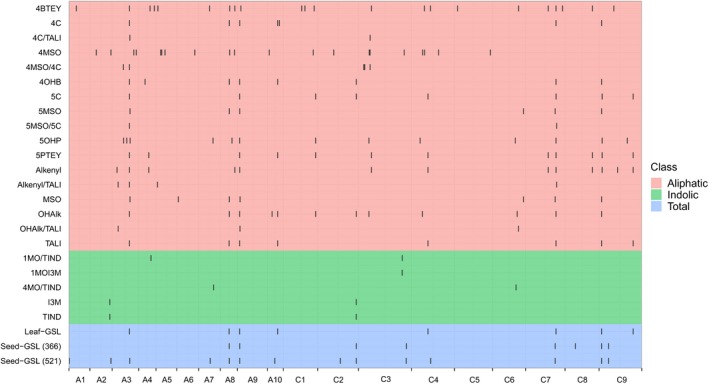
Chromosomal distribution of associated loci for the 25 GSL traits identified in this study. The X‐axis indicates the physical positions of the 19 chromosomes of *B. napus*. The Y‐axis indicates the 25 GSL traits; their abbreviations and descriptions are explained in Table S1. The black vertical line indicates the interval of associated lead SNPs ± 500 kb.

Using 23 426 genome‐wide SNPs with a compressed mixed linear model, we dissected the genetic basis of the variation observed in the 25 GSL traits (one Seed‐GSL trait and 24 GSL traits in the leaves) among the 366 accessions. A total of 177 association signals, corresponding to 78 loci, were significantly associated with one or more GSL traits at a threshold of *P* < 4.27 × 10^−5^ (1/23 426; −log_10_1/23 426 > 4.37) and explained 5.70%–42.60% of the total phenotypic variance (Figure 3, Table S4). All the traits, except 4MOI3M, had at least one significant association, with an average of 3.1 loci per trait.

Thirty‐six *B. napus* candidate genes were homologous to known *A. thaliana* GSL genes (supported with phylogeny tree in Figure S5) located 4.5–834.5 kb away from the lead SNPs, and detailed information on these candidate genes is presented in Table 2. The role of these candidate genes can be divided into five categories: transcription factor (nine genes), side chain elongation (six genes), core structure formation (12 genes), side chain modification (eight genes) and glucosinolate transporter (one gene).

**Table 2:** Candidate genes involved in glucosinolate (GSL) metabolism identified in this study

Candidate gene	Chr.	Position (bp)	Glucosinolate pathway	Orthologous genes in *A. thaliana*
*BnaA02g33530D*	A2	24 045 104–24 047 555	Glucosinolate transporter	*GTR2* §
*BnaA02MYB34 (BnA02g0086460.1)* †	A2	33 020 853–33 024 770†	Transcription factor	*MYB34*
*BnaA03g15200D*	A3	7 021 985–7 023 497	Side chain modification	*GSL‐OH*
*BnaA03g15210D*	A3	7 032 093–7 033 488	Side chain modification	*GSL‐OH*
*BnaA03g25870D*	A3	12 597 840–12 599 565	Side chain modification	*AOP1* §
*BnaA03g35400D*	A3	17 272 166–17 274 784	Side chain elongation	*BCAT4*
*BnaA03g39680D*	A3	19 797 571–19 799 745	Side chain elongation	*MAM1*
*BnaA03g39710D*	A3	19 802 712–19 807 843	Side chain elongation	*MAM1*
*BnaA03g39720D*	A3	19 819 315–19 822 416	Side chain elongation	*MAM1*
*BnaA03g40190D*	A3	20 068 371–20 070 388	Transcription factor	*MYB28*
*BnaA04g24160D*	A4	17 769 754–17 771 713	Core structure formation	*CYP83A1*
*BnaA05g11170D*	A5	6 242 022–6 244 865	Core structure formation	*UGT74C1*
*BnaA06g31890D*	A6	21 347 066–21 349 175	Transcription factor	*MYB118*
*BnaA07g21250D*	A7	16 508 346–16 510 035	Side chain modification	*IGMT5*
*BnaA07g21440D*	A7	16 614 418–16 615 412	Core structure formation	*ST5b*
*BnaA08g16110D*	A8	13 261 309–13 262 552	Core structure formation	*APK2*
*BnaA08g24880D*	A8	17 264 540–17 266 501	Side chain modification	*FMO‐GSOX5*
*BnaA09MYB28* ‡	A9	–	Transcription factor	*MYB28* §
*BnaC01g39030D*	C1	37 815 547–37 818 883	Transcription factor	*IQD1*
*BnaC02g27590D*	C2	25 549 349–25 552 781	Side chain elongation	*MAM1*
*BnaC02g41860D*	C2	44 703 229–44 706 976	Transcription factor	*MYB34*
*BnaC02MYB28* ‡	C2	–	Transcription factor	*MYB28* §
*BnaC03g61420D*	C3	50 525 102–50 527 387	Side chain modification	*CYP81F4*
*BnaC04g12860D*	C4	10 121 243–10 124 859	Core structure formation	*UGT74C1*
*BnaC04g29320D*	C4	30 861 117–30 862 925	Core structure formation	*CYP83A1* §
*BnaC06g24240D*	C6	26 069 733–26 070 781	Core structure formation	*ST5b*
*BnaC06g25040D*	C6	26 610 960–26 611 974	Side chain modification	*CYP81F1*
*BnaC06g25050D*	C6	26 612 805–26 613 371	Side chain modification	*CYP81F1*
*BnaC06g38820D*	C6	36 250 464–36 251 620	Core structure formation	*GSTU20*
*BnaC06g38830D*	C6	36 252 589–36 253 745	Core structure formation	*GSTU20*
*BnaC06g38840D*	C6	36 254 141–36 254 958	Core structure formation	*GSTU20*
*BnaC06g38850D*	C6	36 255 468–36 256 984	Core structure formation	*GSTU20*
*BnaC07MYB28 (BnC07g0816690.1)* †	C7	47 319 003–47 320 378†	Transcription factor	*MYB28* §
*BnaC09g05300D*	C9	3 100 005–3 101 076	Transcription factor	*MYB28* §
*BnaC09g14380D*	C9	10 979 579–10 980 685	Core structure formation	*ST5b*
*BnaC09g23540D*	C9	21 068 937–21 069 920	Side chain elongation	*BAT5* §

^†^ Candidate genes in bracket and their position were based on the *B. napus* ‘ZS11’ reference genome Sun *et al. *(2017).

^‡^ Candidate genes were deleted from low‐glucosinolate accessions according to Harper *et al. *(2012).

^§^ Candidate genes that consistent with Lu *et al. *(2014).

The GSL traits were divided into two biosynthesis groups, aliphatic and indolic GSL traits. Little overlap was observed between the two groups, which is in agreement with the weak correlation between traits in the two groups, reflecting the independence of their biosynthesis pathways. Analysis of the candidate genes revealed that MYB transcription factors were the main genes controlling the biosynthesis of GSLs. Five loci on chromosomes A3, A9, C2, C7 and C9 were identified as the major loci associated with aliphatic GSL content (TALI, 4C and 5C), corresponding to the genes loci of *BnaA03MYB28* (*BnaA03g40190D*), *BnaA09MYB28*, *BnaC02MYB28*, *BnaC07MYB28 (BnC07g0816690.1)* and *BnaC09MYB28* (*BnaC09g05300D*), respectively. Interestingly, all of them are homologous to the *A. thaliana* transcription factor *MYB28*, which positively controls the biosynthesis of aliphatic GSLs (Gigolashvili *et al.*, 2007; Hirai *et al.*, 2007; Sønderby *et al.*, 2007). Two loci were associated with TIND and I3M on chromosomes A2 and C2 (Figure 3, Table S4). *BnA02g0086460.1* and *BnaC02g41860D* are the candidate genes. They are homologous to the *A. thaliana* transcription factor *MYB34*, which positively controls the biosynthesis of indolic GSLs (Celenza *et al.*, 2005).

Based on the GWAS data, we outlined a set of key candidate genes that participate in individual steps of GSL biosynthesis (Figure 1). On chromosome A3, a significant peak at 20.5 Mb associated with 4C/TALI was identified (Table S4). Three candidate genes located in tandem, *BnaA03g39680D*, *BnaA03g39710D* and *BnaA03g39720D*, are orthologous to *A. thaliana* methylthioalkylmalate synthase (*MAM*) genes, controlling the side chain elongation in GSL biosynthesis (Kroymann *et al.*, 2003; Kroymann *et al.*, 2001; Textor *et al.*, 2007). *BnaA03g15200D* and *BnaA03g15210D* are two candidate genes that lie in tandem ~40 kb upstream from the lead SNP Bn‐A03‐p7688578 (associated with OHAlk/TALI), which are orthologous to the *A. thaliana GS‐OH* gene encoding a 2‐oxoacid‐dependent dioxygenase involved in the biosynthesis of hydroxylated alkenyl aliphatic GSLs (Hansen *et al.*, 2008). Similarly, *BnaA08g24880D* is orthologous to *A. thaliana FMO‐GSOX5*, which encodes a flavin monooxygenase involved in the formation of cancer‐preventive S‐oxygenated aliphatic GSLs (Li *et al.*, 2008). *BnaC03g61420D*, *BnaC06g25040D* and *BnaC06g25050D* are orthologous to *A. thaliana CYP81Fs*, which belong to a small subfamily of cytochrome P450 monooxygenase genes whose products catalyse the conversion of I3M to 4‐hydroxy‐indol‐3‐ylmethyl and/or 1‐hydroxy‐indol‐3‐ylmethyl GSL intermediates (Pfalz *et al.*, 2011). Taken together, the analyses (Figure 1, Table 2) provided a framework for the genetic architecture of GSL accumulations in the leaves of *B. napus.*

### Common and tissue‐specific loci in leaves and seeds reveal a complex genetic architecture for GSL accumulations

Phenotypic analysis revealed that Leaf‐GSL and Seed‐GSL were strongly correlated (Figure 2), so the significant associations were compared between these two traits. Eight loci were associated each with Leaf‐GSL (*GSL‐A3*, *GSL‐A8*, *GSL‐A9*, *GSL‐A10‐2*, *GSL‐C4‐1*, *GSL‐C7*, *GSL‐C9‐1* and *GSL‐C9‐3*) and Seed‐GSL (*GSL‐A8*, *GSL‐A9*, *GSL‐C2‐2*, *GSL‐C3*, *GSL‐C7*, *GSL‐C8*, *GSL‐C9‐1* and *GSL‐C9‐2*) and explained 6.11%–42.60% of the total phenotypic variance (Figure 3, Table 3). *GSL‐A9* had the largest effect on both Leaf‐GSL and Seed‐GSL. *GSL‐A8* and *GSL‐C3* are adjacent to *FAE1* (*fatty acid elongase 1*, a key gene involved in the control of erucic acid synthesis) in *B. napus*. A previous study (Howell *et al.*, 2003) did not identify seed GSL QTLs on chromosomes A8 and C3 in a population derived from the cross between *B. napus* cultivar Victor (with high seed GSL content and high seed erucic acid content) and cultivar Tapidor (with low seed GSL content and low seed erucic acid content). When the GWAS was performed with erucic acid content as a covariate, the association signals on chromosomes A8 and C3 were absent (Figure S6). Such a result raised a possibility that *GSL‐A8* and *GSL‐C3* might be false positives due to high correlations between GSL and erucic acid content. *GSL‐C2‐2* was also associated with 4OHB, 5C and OHAlk in the leaves (Table S4).

**Table 3:** Significant associated signals for total glucosinolate (GSL) levels in leaves and seeds by GWAS

Loci	Class	Trait†	Lead SNP	Chr.	Position	MAF (521 lines)	MAF (257 lines)	−log_10_ (P)	PVE (%)‡	Candidate gene
*GSL‐A3*	Common	Leaf‐GSL	Bn‐A03‐p21329715	A3	20 095 857	0.24	0.33	5.91	8.23	*BnaA03g40190D*
		Seed‐GSL (521 lines)	Bn‐A03‐p21669774	A3	20 452 811	0.22	0.20	4.84	4.76	
*GSL‐A9*	Common	Leaf‐GSL	Bn‐A01‐p9004629	A9	2 580 835	0.18	0.00	14.28	20.40	*BnaA09MYB28* ¶
		Seed‐GSL (366 lines)	Bn‐A09‐p2733282	A9	2 677 575	0.22	0.01	25.36	42.60	
		Seed‐GSL (521 lines)	Bn‐A09‐p2733282	A9	2 677 575	0.22	0.01	36.27	44.05	
*GSL‐C2‐2* §	Common	Seed‐GSL (366 lines)	Bn‐scaff_17177_1‐p441984	C2	44 768 013	0.21	0.03	10.19	15.09	*BnaC02MYB28* ¶
		Seed‐GSL (521 lines)	Bn‐scaff_17177_1‐p441984	C2	44 768 013	0.21	0.03	12.81	13.17	
*GSL‐C7*	Common	Seed‐GSL (366 lines)	Bn‐scaff_18181_1‐p1849246	C7	34 322 798	0.19	0.05	6.61	9.37	*BnaC07MYB28 (BnC07g0816690.1)* **
		Seed‐GSL (521 lines)	Bn‐scaff_18181_1‐p1849246	C7	34 322 798	0.19	0.05	9.29	9.25	
		Leaf‐GSL	Bn‐scaff_15705_1‐p2274493	C7	35 279 702	0.18	0.01	6.52	8.84	
*GSL‐C9‐1*	Common	Leaf‐GSL	Bn‐scaff_19783_1‐p379086	C9	2 850 069	0.08	0.01	7.45	11.39	*BnaC09g05300D*
		Seed‐GSL (366 lines)	Bn‐scaff_19783_1‐p379086	C9	2 850 069	0.08	0.01	11.20	17.68	
		Seed‐GSL (521 lines)	Bn‐scaff_19783_1‐p379086	C9	2 850 069	0.08	0.01	11.33	12.13	
*GSL‐A1*	Seed‐specific	Seed‐GSL (521 lines)	Bn‐A01‐p970103	A1	588 064	0.15	0.11	4.53	4.75	
*GSL‐A2*	Seed‐specific	Seed‐GSL (521 lines)	Bn‐A09‐p10577283	A2	24 468 610	0.15	0.07	4.73	5.25	*BnaA02g33530D*
*GSL‐A7*	Seed‐specific	Seed‐GSL (521 lines)	Bn‐A02‐p745468	A7	13 377 023	0.05	0.02	5.89	5.46	
*GSL‐A10‐1*	Seed‐specific	Seed‐GSL (521 lines)	Bn‐A10‐p6896063	A10	8 474 298	0.10	0.06	4.55	4.33	
*GSL‐C2‐1*	Seed‐specific	Seed‐GSL (521 lines)	Bn‐scaff_22749_1‐p67780	C2	26 260 892	0.26	0.22	5.12	5.09	*BnaC02g27590D*
*GSL‐C4‐2*	Seed‐specific	Seed‐GSL (521 lines)	Bn‐scaff_16217_1‐p181427	C4	22 294 107	0.42	0.48	4.73	4.52	
*GSL‐C8*	Seed‐specific	Seed‐GSL (366 lines)	Bn‐A08‐p8426380	C8	11 962 388	0.29	0.12	5.12	7.38	
*GSL‐C9‐2*	Seed‐specific	Seed‐GSL (366 lines)	Bn‐scaff_22835_1‐p619832	C9	11 113 843	0.28	0.38	4.77	6.52	*BnaC09g14380D*
		Seed‐GSL (521 lines)	Bn‐scaff_22835_1‐p619832	C9	11 113 843	0.28	0.38	7.30	7.03	
*GSL‐A10‐2*	Leaf‐specific	Leaf‐GSL	Bn‐A10‐p10454385	A10	11 834 653	0.05	0.02	4.67	6.11	
*GSL‐C4‐1*	Leaf‐specific	Leaf‐GSL	Bn‐scaff_23432_1‐p217818	C4	19 102 451	0.28	0.30	4.50	7.41	
*GSL‐C9‐3*	Leaf‐specific	Leaf‐GSL	Bn‐scaff_17799_1‐p3050608	C9	39 518 182	0.08	0.08	5.73	7.91	

^†^ Leaf‐GSL was anlysed in 366 lines. Seed‐GSL was analysed in 366 lines and 521 lines (indicated in brackets), respectively.

^‡^ Percentage of phenotypic variance explained by lead SNP marker.

^§^ *GSL‐C2‐2* was associated with 4OHB, 5C and OHAlk in the leaves, so it was considered as a common locus.

^¶^ Candidate genes were absent in low‐glucosinolate accessions according to Harper *et al. *(2012).

** Candidate gene in bracket was based on the *B. napus* ‘ZS11’ reference genome (Sun *et al.*, 2017).

To improve the power of GWAS for Seed‐GSL, we performed GWAS on a 521‐member accession panel; there was an association signal at *GSL‐A3*, and additional 6 loci (*GSL‐A1*, *GSL‐A2*, *GSL‐A7*, *GSL‐A10‐1*, *GSL‐C2‐1* and *GSL‐C4‐2*) were identified (Figure 3, Table 3). These results suggest that the genetic basis of the total GSL content in leaves and seeds is quite similar. *GSL‐A3*, *GSL‐A9*, *GSL‐C2‐2*, *GSL‐C7* and *GSL‐C9‐1* were the common loci that controlled the total GSL content in both the leaves and seeds, and the significant SNPs of these loci could explain 61.9% and 81.4% of the total phenotypic variance of the Leaf‐GSL and Seed‐GSL, respectively. *BnaA03MYB28* (*BnaA03g40190D*), *BnaA09MYB28*, *BnaC02MYB28*, *BnaC07MYB28* and *BnaC09MYB28* (*BnaC09g05300D*) were identified as the candidate genes of the 5 loci (Table 3).

On the other hand, some associated loci were not consistent between the Leaf‐GSL and Seed‐GSL. Three loci (*GSL‐A10‐2*, *GSL‐C4‐1* and *GSL‐C9‐3*) were identified to be specifically associated with the Leaf‐GSL in the 366 accessions panel, while eight loci (*GSL‐A1*, *GSL‐A2*, *GSL‐A7*, *GSL‐A10‐1*, *GSL‐C2‐1*, *GSL‐C4‐2*, *GSL‐C8* and *GSL‐C9‐2*) were specifically associated with Seed‐GSL in the 521 accession panel (Table 3). Among them, *GSL‐A2 *was also identified previously (Lu *et al.*, 2014), and the candidate gene *BnaA02g33530D* is homologous to *GTR2*, which encodes a GSL transporter in *A. thaliana* (Nour‐Eldin *et al.*, 2012).

### *GSL‐A3* is associated with higher leaf GSL content in low seed GSL *B. napus*

**Figure 4:**
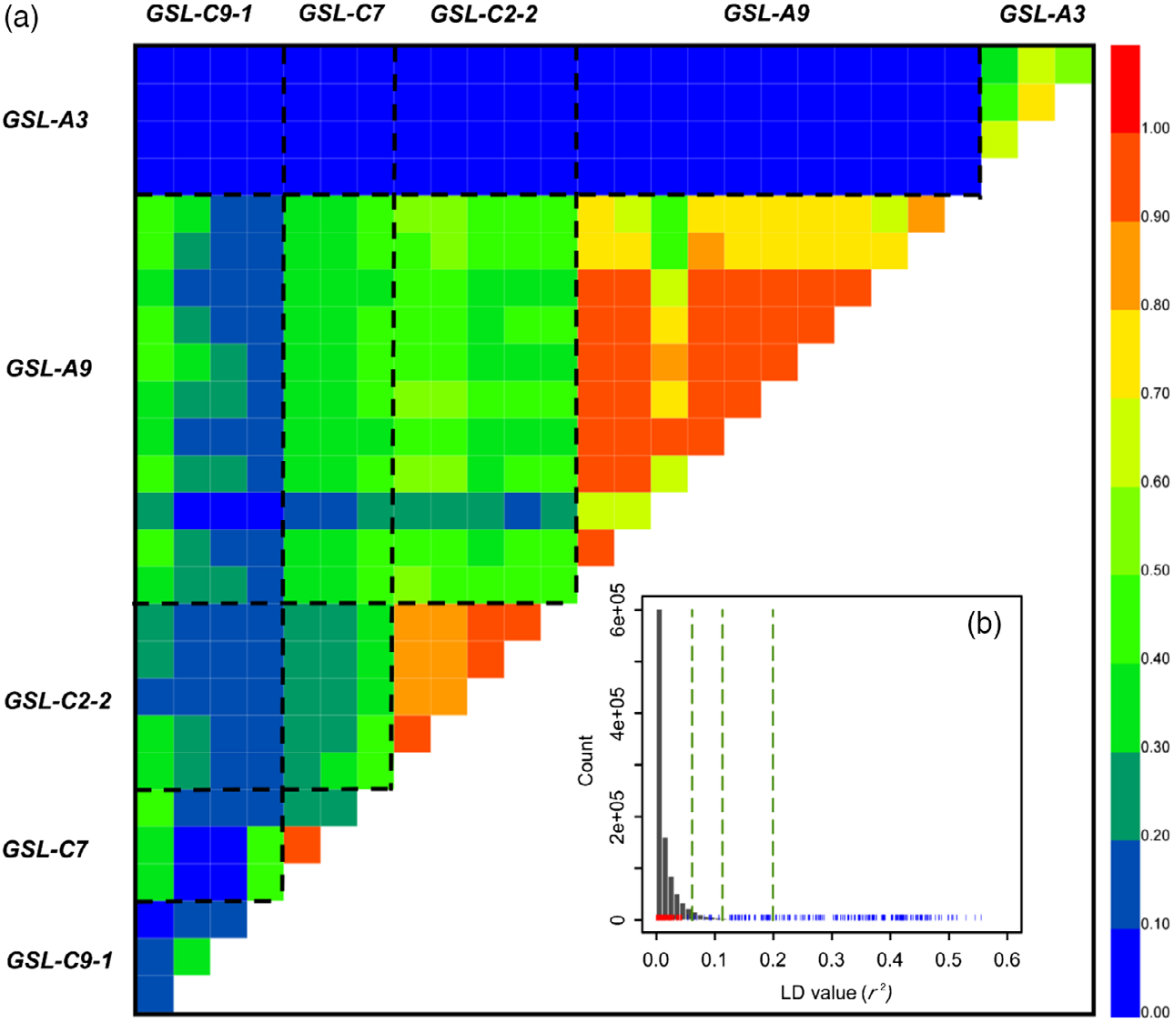
LD among SNPs located within five associated loci: *GSL‐A3*, *GSL‐A9*, *GSL‐C2‐2*, *GSL‐C7* and *GSL‐C9‐1*. (a) Heat map of LD between pairs of SNPs located within five associated loci: *GSL‐A3*, *GSL‐A9*, *GSL‐C2‐2*, *GSL‐C7* and *GSL‐C9‐1*. (b) A null distribution of *r^2^* for 1 000 000 random pairs of SNPs located on different chromosomes (in grey). LD of the associated SNPs within *GSL‐A3* to *GSL‐A9*, *GSL‐C2‐2*, *GSL‐C7* and *GSL‐C9‐1* are indicated in red. LD between the associated SNPs within *GSL‐A9*, *GSL‐C2‐2*, *GSL‐C7* and *GSL‐C9‐1* are indicated in blue. From left to right, the vertical dashed lines mark the 95%, 99% and 99.9% quantiles of the null distribution.

**Figure 5:**
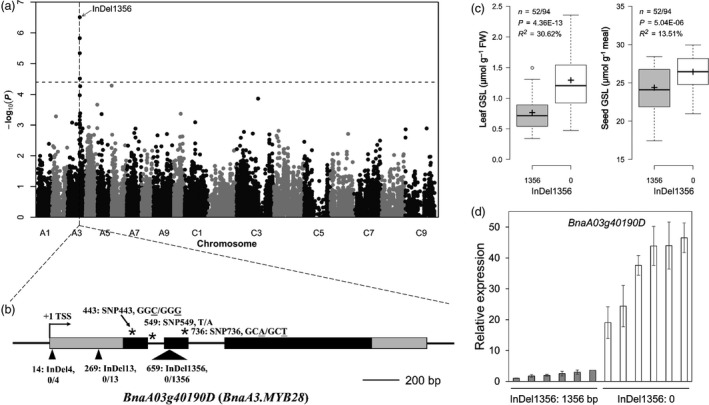
Identified and validated candidate gene *BnaA03g40190D* (*BnaA3.MYB28*) controlling leaf and seed GSL content. (a) Manhattan plot for Leaf‐GSL in a 168‐member accession panel subset with low seed GSL content (Seed‐GSL ≤ 30 µmol/g meal). The association signal of marker InDel1356 is indicated. The dashed horizontal line depicts the uniform significance threshold (−log101/23 429 = 4.37). (b) Gene structure of *BnaA03g40190D* (exons, black boxes; untranslated regions, open boxes) and polymorphism locations (InDels, triangles; SNPs, asterisks). The location of the transcription start site was viewed as + 1, and the location of the other polymorphisms was based on their relative distance from the transcription start site. The SNPs are given in the context of codons; the SNPs are underlined. (c) Box plot for Leaf‐ and Seed‐GSL in the 168‐member accession panel subset, plotted as two alleles of InDel1356: 0‐bp (0) and 1356‐bp (1356) insertions. The *P* value is based on two‐tailed Student’s t tests. *R^2^*, proportion for the phenotypic variation explained by the marker. (d) Bar plots for the mRNA level of *BnaA03g40190D* among 12 accessions with different alleles of InDel1356.

Low seed GSL content is an important breeding goal for rapeseed quality improvement. *GSL‐A3*, *GSL‐A9*, *GSL‐C2‐2*, *GSL‐C7* and *GSL‐C9‐1* are the main loci that control the total GSL content both in leaves and seeds, as mentioned above (Table 3). To evaluate the selection effect during breeding, the minor allele frequency (MAF) of these 5 loci was analysed. The MAF of the lead SNPs of these loci ranged from 0.08 to 0.24 in the 521 accessions panel (Table 3). However, except locus *GSL‐A3*, the lead SNPs of the four loci exhibited a very low allele frequency in a sub‐population of 257 accessions with low seed GSL content (MAF: 0–0.05, Table 3). Linkage disequilibrium (LD) analyses revealed strong, significant genome‐wide correlations between SNPs within the LD block of *GSL‐A9*, *GSL‐C2‐2, GSL‐C7* and *GSL‐C9‐1* (Figure 4a). The strength of these correlations far exceeded that between other pairs of SNPs located on different chromosomes (Figure 4b). Conversely, there were hardly any correlations between *GSL‐A3* and the other 4 loci (Figure 4a)*.* These results suggested that *GSL‐A9*, *GSL‐C2‐2, GSL‐C7* and *GSL‐C9‐1* were co‐selected and fixed during the breeding of double‐low (low seed GSL content, low seed erucic acid content) rapeseed cultivars. In contrast, *GSL‐A3* was not fixed and thus became a major locus associated with Leaf‐GSL variation in the low seed GSL panel. Indeed, when a GWAS for Leaf‐GSL was performed on a 168‐member accession panel from the 366 accessions with low seed GSL content, only *GSL‐A3* was found to be significantly associated via a mixed model (Figure 5a).

### A 1356‐bp insertion in *BnaA3.MYB28* resulted in lower GSL content in leaves

*BnaA03g40190D* is homologous to the *A. thaliana* transcription factor *MYB28* and is located ~27 kb upstream from the lead SNP Bn‐A03‐p21329715 of *GSL‐A3. BnaA03g40190D* is highly similar to *BrA03MYB28* (*Bra012961*) in *B. rapa*; their amino acid sequence similarity is 99.7% (a single amino acid difference, Figure S7). A previous study showed that overexpression of the *BrA03MYB28* gene in *B. rapa* increases the total GSL content in the leaves (Seo *et al.*, 2016). Thus, we deduced that *BnaA03g40190D* is the gene underlying *GSL‐A3.*

To understand how *BnaA03g40190D* affects the GSL content in leaves, the DNA fragments containing the gene were sequenced in 6 accessions with extreme Leaf‐GSL content from the 168‐member accession panel. In total, six polymorphisms were identified throughout a 2112‐bp gene region (Figure 5b). Among these polymorphisms, SNP549 is located in an intron. SNP443 and SNP736 are located in exons, but they did not cause amino acid changes. It is unlikely that those three polymorphisms could lead to a functional differentiation at this locus. To assign the potential functional link to other three mutations, InDel4, InDel13 and InDel1356, between the alleles, we developed PCR‐based markers and genotyped the 168‐member accession panel subsequently with the markers. Interestingly, only InDel1356 was significantly associated with Leaf‐GSL using a mixed model (−log_10_ *P*> 4.37, Figure 5a). The Leaf‐GSL and Seed‐GSL of 52 accessions that had the 1356‐bp insertion were significantly lower than other 94 accessions that lacked the 1356‐bp insertion (*P* < 0.001, Figure 5c). The InDel1356 polymorphism explained 30.62% of the total phenotypic variance in leaves and 13.51% in seeds (Figure 5c). Such a polymorphism resulted from a 1356‐bp insertion in low‐GSL accessions compared with the high‐GSL ones within the second exon, which lead to both a frame shift and a premature stop codon in the putative protein (Figure S8).

Expression analysis revealed that the *BnaA03g40190D* expression level was significantly greater in the six lines lacking the 1356‐bp insertion than that in the lines containing the 1356‐bp insertion (Figure 5d). Taken together, these results thus demonstrated mutation in *BnaA03g40190D* affected the GSL content by regulating gene expression.

## Discussion

Glucosinolates have obtained status as model secondary metabolites, and GSL biosynthesis genes in *A. thaliana* have been successfully characterized via map‐based cloning, mutant collections and co‐expression networks (Sønderby *et al.*, 2010). However, very limited genetic and metabolomic information on GSL biosynthesis in *B. napus* is available. Due to genomic triploidization as well as chromosomal rearrangement, fusion and deletion in *Brassica*, 1–12 copies have been reported in *B. napus* for single‐gene locus in *Arabidopsis* (Tadege *et al.*, 2001). Therefore, large‐scale identification of key genes involved in GSL biosynthesis underlying natural variation is much more complicated in *B. napus*. Recently, GWASs coupled with metabolomics analyses have been carried out in *A. thaliana*, maize and rice to understand the genetics contributions to metabolic diversity (Chan *et al.*, 2011; Chan *et al.*, 2010; Chen *et al.*, 2014; Deng *et al.*, 2017; Wen *et al.*, 2014). Thus, it is possible to screen a large number of accessions simultaneously to identify key genes involved in individual steps of GSL biosynthesis in *B. napus* based on prior knowledge of GSL metabolism in *A. thaliana*. The power to resolve associated loci for a particular trait using GWAS depends on the marker density. In our previous study (Liu *et al.*, 2016), it was indicated that use of ~20 000 SNPs with marker density 1 SNP every 24.9 kb was sufficient to perform a GWAS in *B. napus*, considering the status of LD in our panel of accessions (LD decay in the A and C sub‐genomes was 0.10–0.15 Mb and 1.15–1.20 Mb, respectively). In the present study, using GWAS methodology, we investigated variation in 25 GSL traits among 366 various accessions and identified 36 key genes involved in GSL biosynthesis (Table 2). In addition to nine genes identified in previous association studies, the others were considered newly identified genes that participate in GSL biosynthesis in *B. napus*. The newly identified genes will need more detailed molecular validation to better reveal the complex control of GSL in the allotetraploid species. The combination of GWAS methodology and metabolomics analysis reported here provided an effective way for the large‐scale identification of GSL‐related genes in *B. napus*, and this combination could also apply to other *Brassica* species.

The genetic architecture for GSL is more complex in *B. napus*. In the present study, we identified 78‐associated loci and 36 key genes involved in GSL biosynthesis. However, there were only three known loci *GS‐OH*, *AOP* and *MAM* responsible for natural variation of GSL profile in *A. thaliana* (Brachi *et al.*, 2015; Chan *et al.*, 2011), in which hundreds of genes in GSL pathway were identified. We have observed long‐distance LD at four loci, *BnaA09MYB28*, *BnaC02MYB28*, *BnaC07MYB28* and *BnaC09MYB28* due to artificial selection during double‐low rapeseed breeding. In *A. thaliana*, natural selection was observed to shape natural variation in GSL profiles to adapt to herbivore resistance, leading to long‐distance LD at *GS‐OH* and *MAM* loci. Together, these results indicated that both artificial selection and natural selection have played a role in altering natural variation in GSL.

The different bioactivities of GSLs and their degradation products depend on the structure of the side chain. Controlling the levels of specific GSLs is of considerable interest. The genes and loci identified here provide new insight into the GSL biosynthesis pathway in *B. napus*, which will facilitate the genetic manipulation and metabolic engineering of desirable GSLs in response to changing herbivory or other selective pressures. For example, increased levels of GSLs could reduce the extent of grazing by birds and slugs (Giamoustaris and Mithen, 1995), and increased aliphatic GSLs reduced the extent of generalist pest feeding (Beekwilder *et al.*, 2008). Furthermore, elevated indolic GSLs were found to enhance plant resistance against *Sclerotinia sclerotiorum* and aphids (Pfalz *et al.*, 2009; Stotz *et al.*, 2011; Wu *et al.*, 2016; Zhang *et al.*, 2015), and increased the hydroxylation of butenyl GSLs reduced the extent of adult flea beetle feeding (Giamoustaris and Mithen, 1995).

The introduction of low‐GSL cultivars was accompanied by concerns that these cultivars would be more susceptible to pests and diseases due to the potential protective effects of GSLs. Indeed, increasing bird damage in double‐low rapeseed in China has been a new challenge for growers (Zhao *et al.*, 2016). Although significant variation in leaf GSL content within double‐low breeding lines and cultivars were reported (Beckmann *et al.*, 2007), the genetic basis underlying such variations remains unclear. In the present study, we screened the seed and leaf GSL variation among a large panel and observed that the seed and leaf GSL content were highly correlated (*r* = 0.79; Figure 2c), which is consistent with previous studies, in which moderate to strong correlations between GSL content in the same two tissues were reported (Beckmann *et al.*, 2007; Schilling and Friedt, 1991). This high positive correlation between GSL in seed and leaf is due to the common loci that controlled GSL biosynthesis. We identified five common loci (*GSL‐A3*, *GSL‐A9*, *GSL‐C2‐2*, *GSL‐C7* and *GSL‐C9‐1*) that were associated with leaf and seed GSL content, the different copies of *MYB28* transcription factor were the genes underlying these loci (Table 3). Among these loci, *GSL‐A9*, *GSL‐C2‐2*, *GSL‐C7* and *GSL‐C9‐1* were reported previously (Harper *et al.*, 2012; Li *et al.*, 2014; Lu *et al.*, 2014), while *GSL‐A3* was a novel locus.

According to the DNA polymorphism results and gene expression analysis, *BnaA03g40190D*, which is homologous to the *A. thaliana MYB28* transcription factor that positively controls the biosynthesis of aliphatic GSLs, was considered as the causal gene underlying the *GSL‐A3* locus. *BnaA03MYB28* (*BnaA03g40190D*) regulated leaf and seed GSL content simultaneously, but the effect on leaves is stronger than on seeds. Unlike *BnaA09MYB28*, *BnaC02MYB28*, *BnaC07MYB28* and *BnaC09MYB28*, *BnaA03MYB28* (*BnaA03g40190D*) had minor effect on seeds, which escaped from artificial selection during double‐low rapeseed breeding. Therefore, it was not fixed in a double‐low panel and could be used for high leaf/low seed GSL breeding.

We also identified 3 and 8 tissue‐specific loci in the leaves and seeds, respectively (Table 3). These loci may be associated with tissue‐specific biosynthesis or transport of GSLs. In support of this fact, the candidate gene *BnaA02g33530D* is homologous to *GTR2*, which encodes a GSL transporter in *A. thaliana* (Nour‐Eldin *et al.*, 2012). Associative transcriptomic analyses have revealed that the expression levels of *BnaA02g33530D* in juvenile leaves are correlated with the accumulation of GSLs in seeds (Lu *et al.*, 2014). In a particular low‐GSL *B. napus* breeding line, there are 12 GTR1 and GTR2 functional transporters (Nour‐Eldin *et al.*, 2017). Engineering GSL transporters by molecular marker‐assisted selection, mutation and genome editing could be an effective way for breeding of high leaf/low seed GSL content. Recently, Nour‐Eldin *et al. *(2017) mutated one of seven and four of 12 GTR orthologs, which reduced GSL levels in seeds by 60%–70% in two different *Brassica* species (*B. rapa* and *B. juncea*). Additional validation and cloning of the tissue‐specific loci will help understand the molecular basis of leaf and seed GSL metabolism and thus further facilitate the breeding of high leaf/low seed GSL content.

## Experimental procedures

### Plant materials and growing environments

The association panel used in this study consisted of 521 *B. napus* accessions, and detailed information on these lines can be found in a previous study (Liu *et al.*, 2016). The association panel was cultivated at the experimental farm of Huazhong Agricultural University, Wuhan, China, for three consecutive years (2012–2013, 2013–2014 and 2014–2015). The field experiments followed a randomized complete block design with two or three replications (2014–2015, only two replications). Each accession was grown in a plot; there were 10–12 plants each in two rows, and the distance between plants was 21 cm within each row and 30 cm between rows. The trial management was in accordance with standard breeding field protocols.

### Analysis of GSL content

Three hundred and sixty‐six accessions, which composed a subset of an association panel, were selected from 521 initial accessions. The GSL content of the excised leaves of these 366 accessions grown in two consecutive years (2013–2014 and 2014–2015) was analysed; two biological replications were included. At approximately 90 days after sowing, when the plants had eight true leaves and before bolting, the third leaf from the top was harvested from 2–3 plants of each plot and combined to represent one biological replication. The combined sample was placed into a 50‐mL tube that contained 25 mL of 90% methanol and twenty 3‐mm stainless steel ball bearings. GSLs were extracted from the leaves and analysed via HPLC in accordance with previously described methods, with modifications (Kliebenstein *et al.*, 2001b). The tissue was homogenized for ~10 min in a paint shaker and then centrifuged, after which the supernatant was transferred to a 12‐mL column that contained 1 mL of DEAE Sephadex A‐25. The Sephadex‐bound GSLs were eluted by incubation with sulphatase. Individual desulpho‐GSL compounds were identified by their retention times and quantified using 2‐propenyl GSL (Sinigrin, Sigma‐Aldrich) as an internal standard as described by Feng *et al. *(2012) and Zhang *et al. *(2015). The results were expressed as µmol/g FW. In addition to the content of individual GSLs, we used a set of summation and ratio traits based on prior knowledge of GSL pathways in *A. thaliana* to examine the variation at individual steps of GSL biosynthesis (Table S1).

The total GSL content of seeds produced during in two consecutive years (2012–2013 and 2013–2014) was analysed; three replications were included in each year. At maturity, five representative plants in the middle of each plot were harvested. The total GSL content of the dried seeds was determined via a Foss NIRSystems 5000 near‐infrared reflectance spectroscope and expressed as µmol/g meal.

### Statistical analysis

The broad‐sense heritability was calculated as $H^{2}=\sigma_{g}^{2}/\left(\sigma_{g}^{2}+\frac{\sigma_{\text{ge}}^{2}}{n}+\frac{\sigma_{e}^{2}}{\mathrm{nr}}\right)$, where $\sigma_{g}^{2}$ is the genetic variance, $\sigma_{\text{ge}}^{2}$ is the interaction variance of the genotype by environment, $\sigma_{e}^{2}$ is the error variance, *n* is the number of environments and *r* is the number of replications. The estimates of $\sigma_{g}^{2}$, $\sigma_{\text{ge}}^{2}$ and $\sigma_{e}^{2}$ were obtained from the ANOVA procedure in SAS 9.3 (SAS Institute, Inc., Raleigh, NC). The best linear unbiased predictions (BLUPs) for each line across the two environments were calculated with the MIXED procedure in SAS 9.3 and were used for evaluating trait variation and for the association analysis.

### Genotyping and quality control

Genotyping of the 521 accessions was performed with *Brassica* 60K Illumina Infinium^®^ HD Assay SNP arrays (Illumina Inc., San Diego, CA) in accordance with the manufacturer’s protocol. For quality control, SNPs with an AA or BB frequency equal to zero, a missing rate >0.2, a heterozygous rate >0.2 and a MAF ≤ 0.05 were excluded. For the physical localization of SNP markers, the probe sequences of 52 157 SNPs were used to perform a BlastN (Altschul *et al.*, 1990) query against the *B. napus* ‘Darmor‐*bzh*’ reference genome version 4.1 (Chalhoub *et al.*, 2014). Only, the top BLAST hits in accordance with an e‐value threshold of e‐10 were considered mapped in the genome. SNPs with multiple or unknown chromosomal locations were also excluded. After the filtering process was performed, 521 accessions with 23 168 SNPs and 366 accessions with 23 426 SNPs remained for further analysis.

### Genome‐wide association analysis

We performed population structure and genetic relatedness analyses using a subset of 4107 SNPs that were distributed evenly (one SNP every 100 kb) across the entire genome. The model‐based program STRUCTURE v2.3.4 (Pritchard *et al.*, 2000) was used to infer the population structure. The number of subgroups (K) was set from 1 to 5. Three runs for each K were performed using the admixture and correlated allele frequencies model; the burn‐in length and iterations both were set to 100 000. The method described by Evanno *et al. *(2005) was used to estimate the optimal K value. A kinship matrix was subsequently calculated to estimate the pairwise relatedness between individuals via the software package SPAGeDi (Hardy and Vekemans, 2002).

Trait‐SNP associations were assessed using a compressed mixed linear model that accounted for population structure and relative kinship via the program TASSEL 4.0 (Bradbury *et al.*, 2007; Yu *et al.*, 2006; Zhang *et al.*, 2010). The significance of associations between SNPs and the traits was based on a uniform threshold (*P* < 1/*n*, *n* = total numbers of markers used). Using the lm function in R software, we then performed stepwise regression to estimate the phenotypic variation explained by multiple SNPs (Ihaka and Gentleman, 1996).

The candidate genes were screened surrounding the associated loci according to known *A. thaliana* GSL genes and supported with phylogeny tree. Phylogeny tree was analysed by MAFFT tool (multiple alignment using fast Fourier transform, http://www.ebi.ac.uk/Tools/services/web/toolform.ebi?tool=mafft).

### Linkage disequilibrium

The LD between SNPs was estimated by the *r^2^* parameter calculated with the program TASSEL 4.0. Only, homozygous SNPs were used; heterozygous SNPs were set to missing. LD blocks were defined for each associated locus when flanking markers presented strong LD (*r* ^2^ > 0.4) with a lead SNP, as described by Hatzig *et al. *(2015).

### *BnaA03g40190D* resequencing and analysis

The genomic sequence of *BnaA03g40190D* was used to design primers via Primer Premier 5. Primer pairs BnA3.MYB28‐1 and BnA3.MYB28‐2 were used to amplify and sequence *BnaA03g40190D* in six accessions that presented extreme Leaf‐GSL (GenBank accession numbers MN103513‐MN103518). The sequences were aligned with Clustal Omega (://www.ebi.ac.uk/Tools/msa/clustalo/) and refined manually with BioEdit. The gene structure was predicted by Fgenesh (Softberry, Inc., Mount Kisco, NY), and its full‐length coding sequence (CDS) was confirmed by sequencing using synthesized cDNA as a template. Three *BnaA03g40190D* InDels (InDel4, InDel13 and InDel1356) among a 168‐member accession panel subset with low seed GSL content were genotyped with PCR‐based markers. The primers used in this experiment are listed in Table S5.

### Gene expression analysis

For quantitative RT‐PCR (qRT‐PCR) analysis, leaves were collected at approximately 90 days after sowing. Their total RNA was isolated with TRIzol Reagent (Invitrogen, Carlsbad, CA), after which 2 µg was used for synthesizing cDNA with a Thermo Scientific RevertAid First Strand cDNA Synthesis Kit. The qRT‐PCR included both three biological replicates and three technical replicates and was performed via a Bio‐Rad CFX96 Real‐Time system (Bio‐Rad, Hercules, CA) in conjunction with a DBI Bioscience Bestar Real‐Time PCR Master Mix kit in accordance with the manufacturers’ instructions. The data were analysed with LINREG as described by Ramakers *et al. *(2003). The primers used in this experiment are listed in Table S5.

## Conflict of interest

The authors declare that they have no conflicts of interest.

## Author contributions

YZ conceived the study. SL and YZ designed the experiments. SL and CF performed the genotyping of the association panel. SL, HH, XY and YZ participated in the phenotyping of the GSL traits. SL and HH performed the gene cloning and expression analysis. SL, QY, CZ and YZ analysed the data. SL, HH and YZ wrote the paper. SL and HH contributed equally to the work. All the authors have read and approved the manuscript.

## Supporting information


**Figure S1** Distribution of 4OHB, 4MSO, 4BTEY, 5OHP, 5MSO and 5PTEY in leaves.
**Figure S2** Distribution of I3M, 4MOI3M, 1MOI3M, Leaf‐GLS, TALI and TIND in leaves.
**Figure S3** Distribution of I3M, 4MOI3M, 1MOI3M, Leaf‐GLS, TALI and TIND in leaves.
**Figure S4** Distribution of 5MSO/5C, 4C/TALI, OHAlk/TALI, Alkenyl/TALI, 4MO/TIND and 1MO/TIND in leaves.
**Figure S5** Phylogenetic tree of 35 candidate gene in *B. napus* and their Arabidopsis orthologue genes.
**Figure S6** Manhattan plot of the total seed glucosinolate (GSL) content (Seed‐GSL) among the 366‐member accession panel; erucic acid content served as the covariate.
**Figure S7** Manhattan plot of the total seed glucosinolate (GSL) content (Seed‐GSL) among the 366‐member accession panel; erucic acid content served as the covariate.
**Figure S8** Manhattan plot of the total seed glucosinolate (GSL) content (Seed‐GSL) among the 366‐member accession panel; erucic acid content served as the covariate.
**Table S1** Abbreviations and descriptions of glucosinolate (GSL) traits in this study.
**Table S2** Correlations among the 25 glucosinolate (GSL) traits.
**Table S3** Correlations among the 25 glucosinolate (GSL) traits. Correlations among the 25 glucosinolate (GSL) traits.
**Table S4** Summary of significant genome‐wide association signals for the 25 glucosinolate (GSL) traits.
**Table S5** Primers used in this study.
