# Supplementary material for: Dissection of genetic architecture for glucosinolate accumulations in leaves and seeds of Brassica napus by genome‐wide association study
Source: Plant Biotechnol J. 2019 Dec 25;18(6):1472–84. doi: 10.1111/pbi.13314 (PMC7206990; doi:10.1111/pbi.13314)
Supplement: Supplementary file 1 — Figure S1 Distribution of 4OHB, 4MSO, 4BTEY, 5OHP, 5MSO and 5PTEY in leaves. Figure S2 Distribution of I3M, 4MOI3M, 1MOI3M, Leaf‐GLS, TALI and TIND in leaves. Figure S3 Distribution of I3M, 4MOI3M, 1MOI3M, Leaf‐GLS, TALI and TIND in leaves. Figure S4 Distribution of 5MSO/5C, 4C/TALI, OHAlk/TALI, Alkenyl/TALI, 4MO/TIND and 1MO/TIND in leaves. Figure S5 Phylogenetic tree of 35 candidate gene in B. napus and their Arabidopsis orthologue genes. Figure S6 Manhattan plot of the total seed glucosinolate (GSL) content (Seed‐GSL) among the 366‐member accession panel; erucic acid content served as the covariate. Figure S7 Manhattan plot of the total seed glucosinolate (GSL) content (Seed‐GSL) among the 366‐member accession panel; erucic acid content served as the covariate. Figure S8 Manhattan plot of the total seed glucosinolate (GSL) content (Seed‐GSL) among the 366‐member accession panel; erucic acid content served as the covariate. Table S1 Abbreviations and descriptions of glucosinolate (GSL) traits in this study. Table S2 Correlations among the 25 glucosinolate (GSL) traits. Table S3 Correlations among the 25 glucosinolate (GSL) traits. Correlations among the 25 glucosinolate (GSL) traits. Table S4 Summary of significant genome‐wide association signals for the 25 glucosinolate (GSL) traits. Table S5 Primers used in this study. [file PBI-18-1472-s001.pdf]

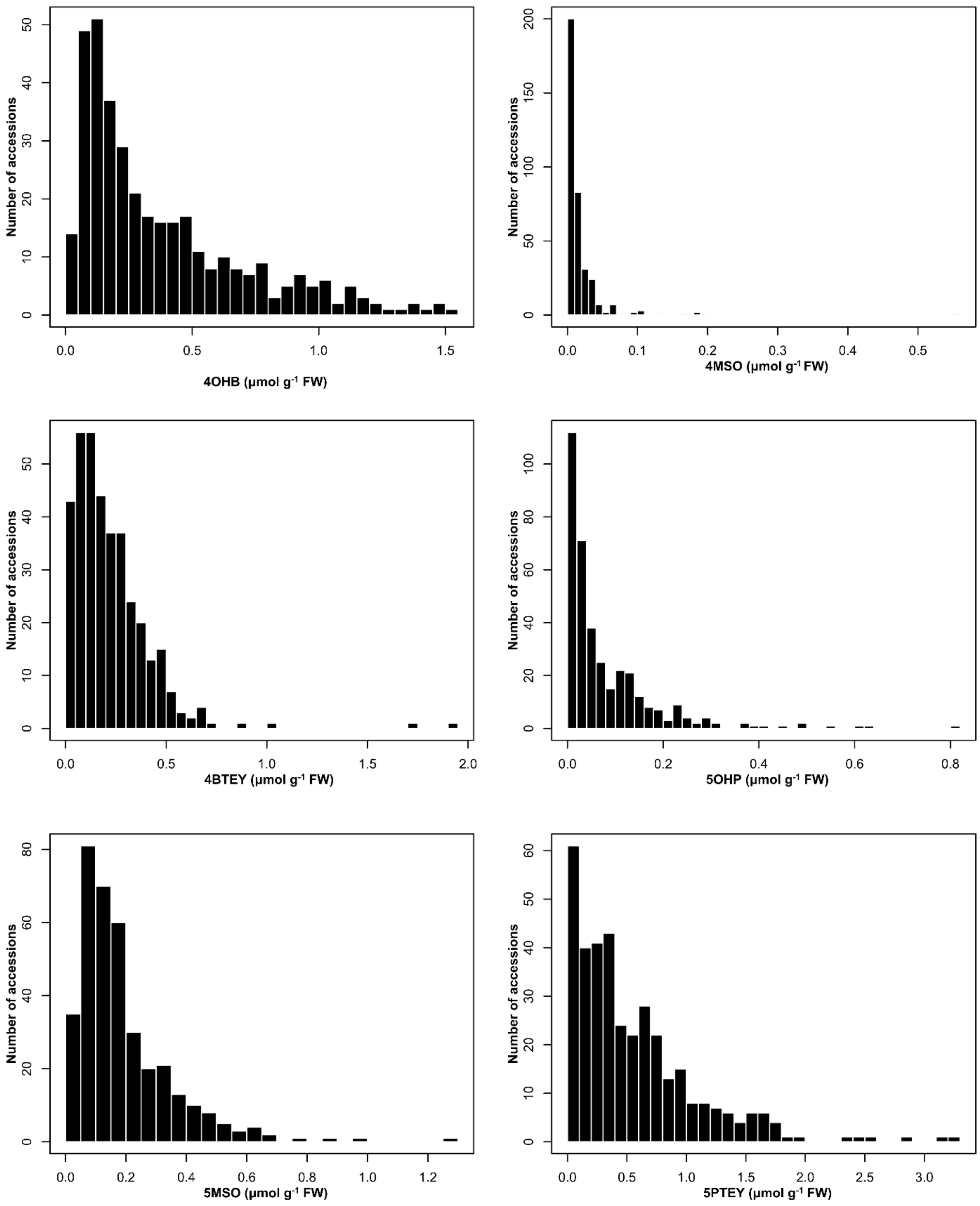

Figure S1 Distribution of 4OHB, 4MSO, 4BTEY, 5OHP, 5MSO, 5PTEY in leaves.

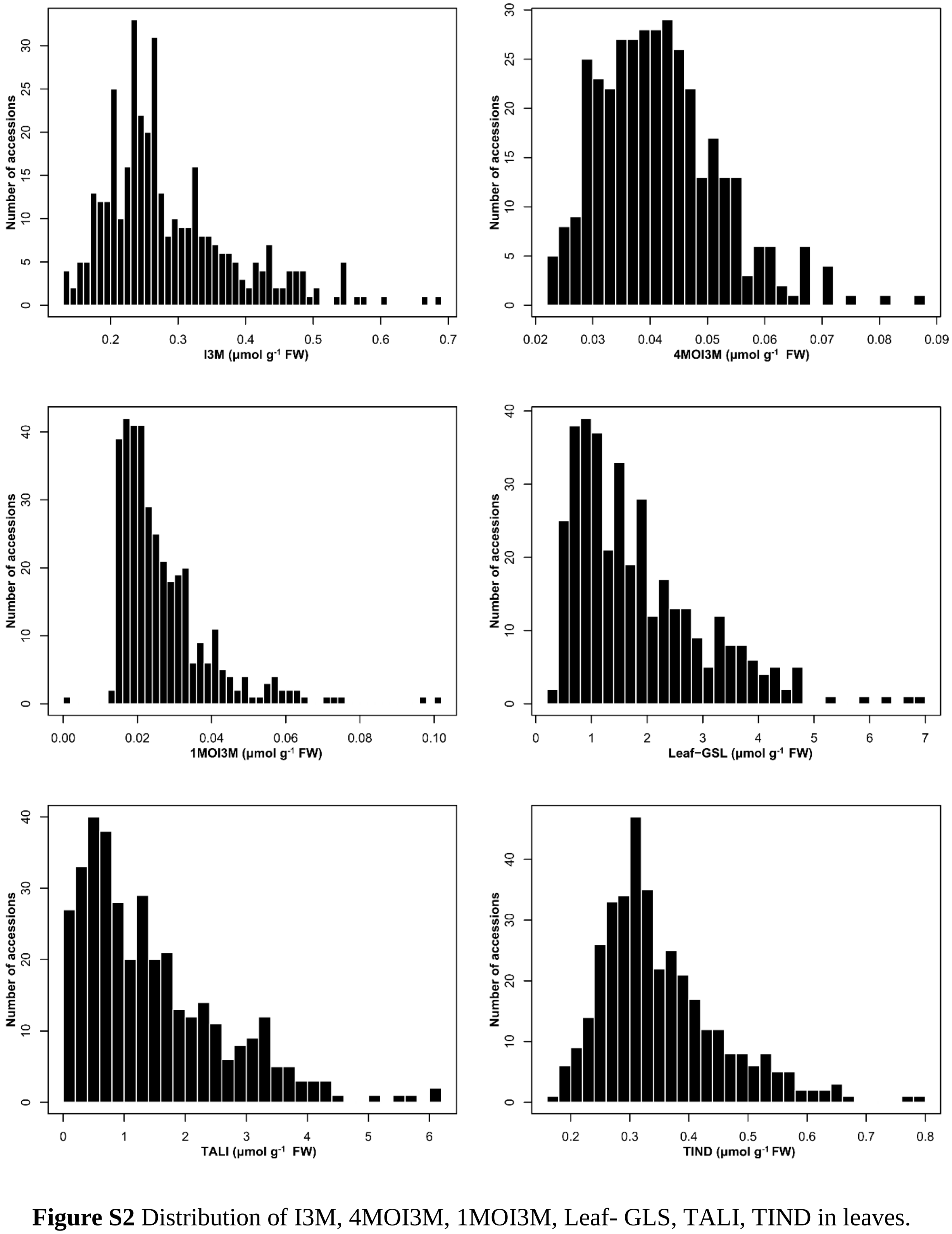

Figure S2 Distribution of I3M, 4MOI3M, 1MOI3M, Leaf- GLS, TALI, TIND in leaves.

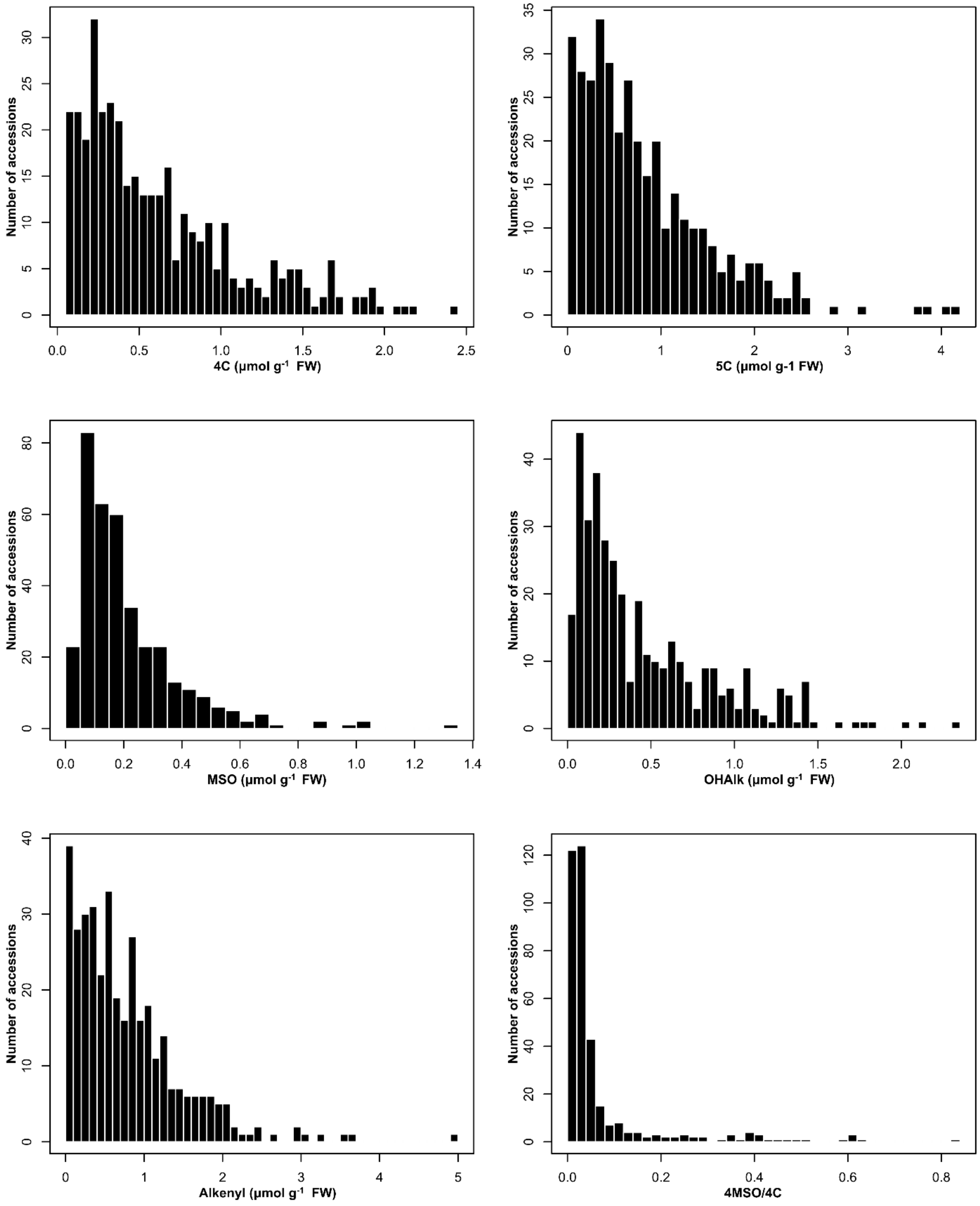

Figure S3 Distribution of 4C, 5C, MSO, OHAlk, Alkenyl, 4MSO/4C in leaves.

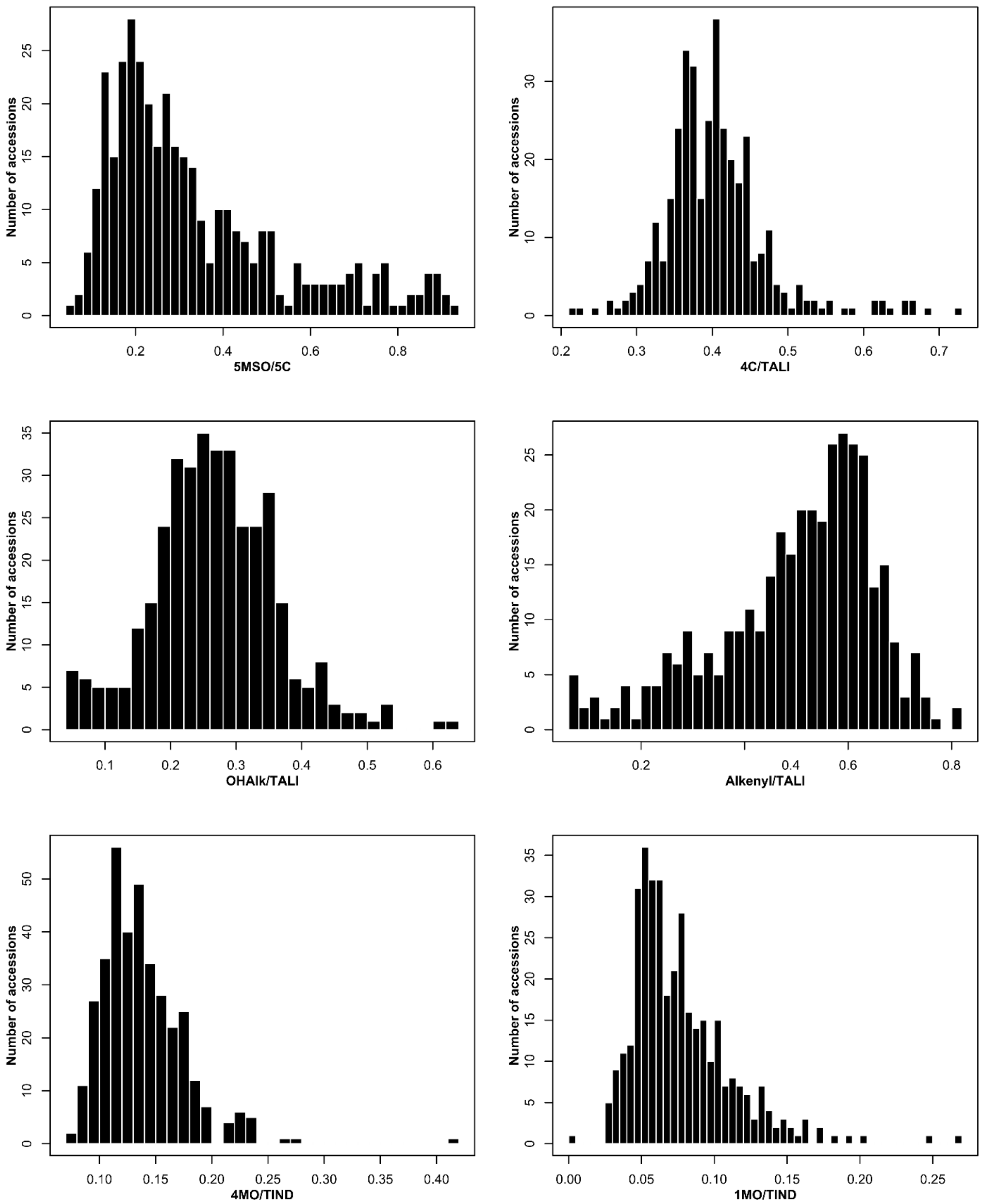

Figure S4 Distribution of 5MSO/5C, 4C/TALI, OHAlk/TALI, Alkenyl/TALI, 4MO/TIND, 1MO/TIND in leaves.

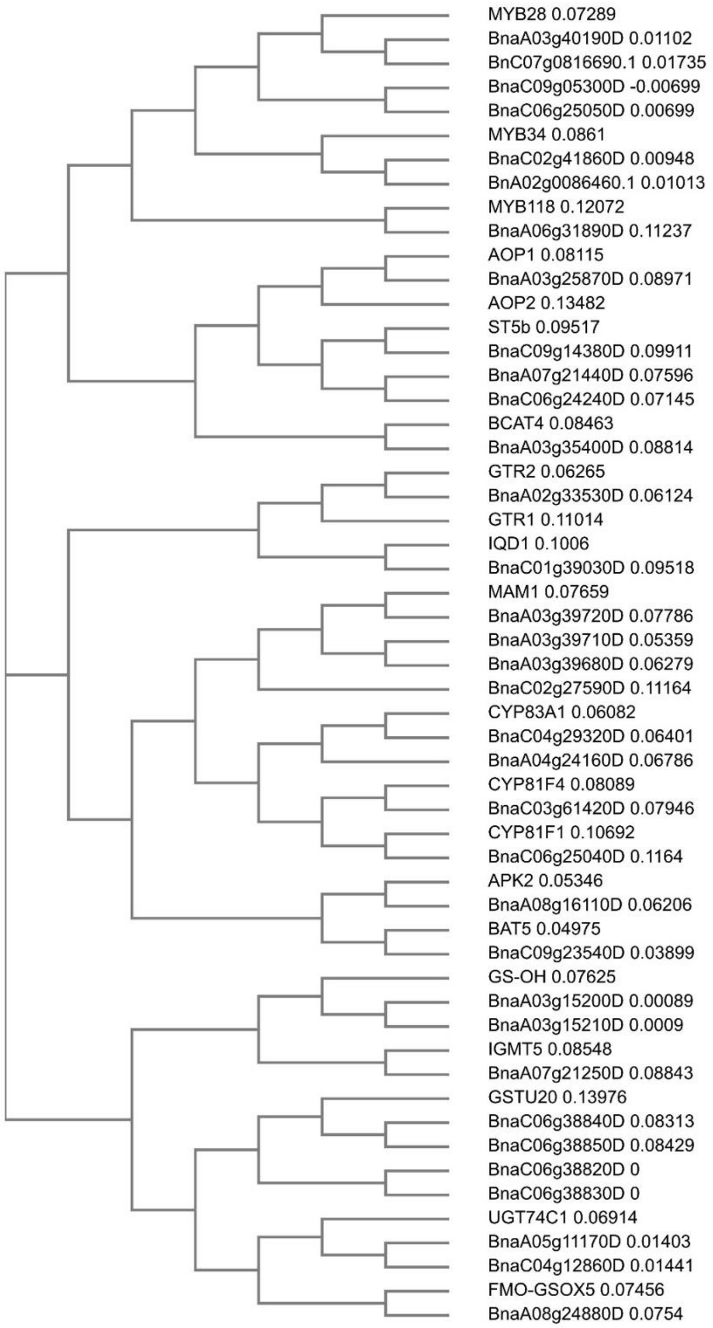

Figure S5 Phylogenetic tree of 35 candidate gene in *B.napus* and their Arabidopsis orthologue genes.

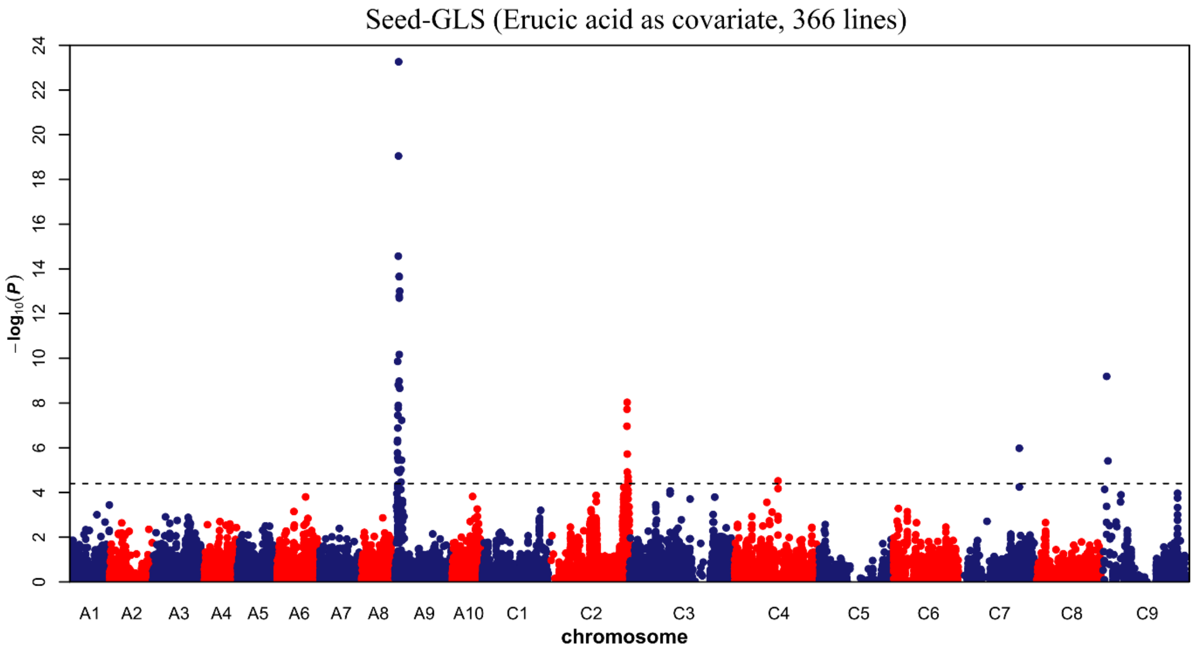

Figure S6 Manhattan plot of the total seed glucosinolate (GSL) content (Seed-GSL) among the 366-member accession panel; erucic acid content served as the covariate.

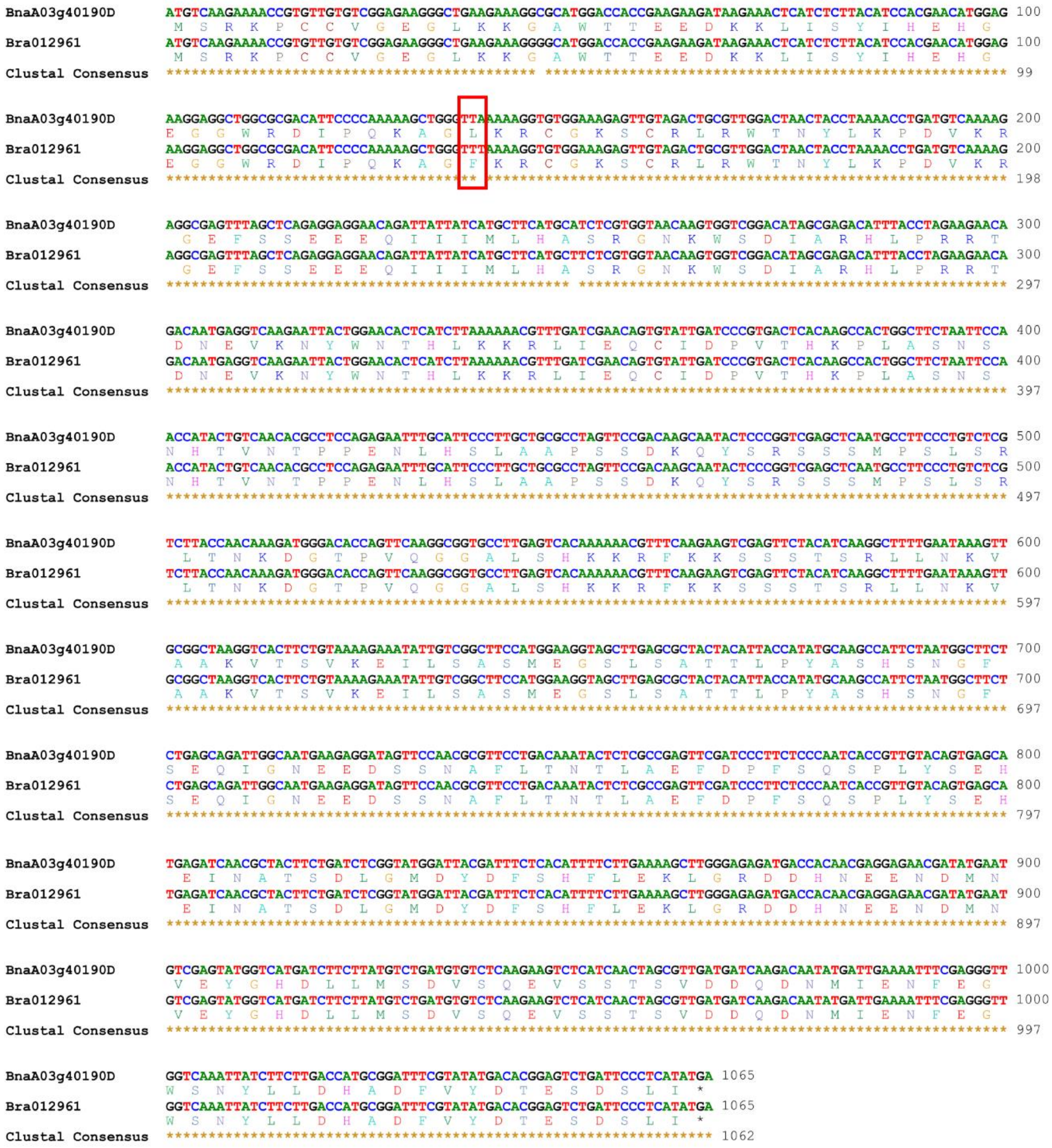

Figure S7 Alignment of the gene sequences of *BnaA03g40190D* and *Bra012961*. The putative protein sequences are given below the nucleotide sequences.

```
InDel1356:1356     1   MSRKPCCVGEGLKKGAWTTEEDKKLISYIHEHGEGGWRDIPQKAGLKRCGKSCRLRWTKSTIIPVSVMGF------------------------------ 70
InDel1356:0        1   MSRKPCCVGEGLKKGAWTTEEDKKLISYIHEHGEGGWRDIPQKAGLKRCGKSCRLRWTNYLKPDVKRGEFSSEEEQIIMLHASRGNKWSDIARHLPRRT 100
Clustal Consensus  1   **********************************************************:     *.   *                              61

InDel1356:1356     70  ---------------------------------------------------------------------------------------------------- 70
InDel1356:0        101 DNEVKNYWNTHLKKRLIEQCIDPVTHKPLASNSNHTVNTPPENLHSLAAPSSDKQYSRSSSMPSLSRLTNKDGTPVQGGALSHKKRFKKSSSTSRLLNKV 200
Clustal Consensus

InDel1356:1356     70  ---------------------------------------------------------------------------------------------------- 70
InDel1356:0        201 AAKVTSVKEILSASMEGSLSATTLPYASHSNGFSEQIGNEEDSSNAFLTNTLAEFDPFSQSPLYSEHEINATSDLGMDYDFSHFLEKLGRDDHNEENDMN 300
Clustal Consensus

InDel1356:1356     70  ------------------------------------------------------ 70
InDel1356:0        301 VEYGHDLLMSDVSQEVSSTSVDDQDNMIENFEGWSNYLLDHADFVYDTESDSLI 354
Clustal Consensus
```

Figure S8 Alignment of the putative protein sequences of two alleles of *BnaA03g40190D* with 0- and 1,356-bp insertions.

Table S1 Abbreviations and descriptions of glucosinolate (GSL) traits in this study.

Abbreviation	GSL type	GSL name or description
4OHB	Aliphatic 4C	2-hydroxy-3-butenyl GSL
4MSO	Aliphatic 4C	4-methylsulfinylbutyl GSL
4BTEY	Aliphatic 4C	3-butenyl GSL
5OHP	Aliphatic 5C	2-hydroxy-4-pentenyl GSL
5MSO	Aliphatic 5C	5-methylsulfinylpentyl GSL
5PTEY	Aliphatic 5C	4-pentenyl GSL
I3M	Indolic	indol-3-ylmethyl GSL
4MOI3M	Indolic	4-methoxy-indol-3-ylmethyl GSL
1MOI3M	Indolic	1-methoxy-indol-3-ylmethyl GSL
Leaf-GSL		Total GSL in leaves
Seed-GSL		Total GSL in seeds
TALI		Total Aliphatic GSL in leaves
TIND		Total Indolic GSL in leaves
4C		Total 4C Aliphatic in leaves
5C		Total 5C Aliphatic in leaves
MSO		Total Methylsulfinyl Aliphatic in leaves
OHAlk		Total Hydroxyalkenyl Aliphatic in leaves
Alkenyl		Total Alkenyl Aliphatic in leaves
4MSO/4C		Ratio of 4MSO to TALI in leaves
5MSO/5C		Ratio of 5MSO to TALI in leaves
4C/TALI		Ratio of 4C to TALI in leaves
OHAlk/TALI		Ratio of OHAlk to TALI in leaves
Alkenyl/TALI		Ratio of Alkenyl Aliphatic to TALI in leaves
4MO/TIND		Ratio of 4MOI3M to TIND in leaves
1MO/TIND		Ratio of 1MOI3M to TIND in leaves

Table S2 Correlations among the 25 glucosinolate (GSL) traits.

Trait	4OHB	4MSO	4BTEY	5OHP	5MSO	5PTEY	I3M	4MOI3M	1MOI3M	Leaf-GSL	Seed-GSL	TALI	TIND	4C	5C	MSO	OHAlk	Alkenyl	4MSO/4C	5MSO/5C	4C/TALI	OHAlk/TALI	Alkenyl/TALI	4MO/TIND
4MSO	0.20**																							
4BTEY	0.84**	0.24**																						
5OHP	0.89**	0.06	0.63**																					
5MSO	0.76**	0.56**	0.73**	0.63**																				
5PTEY	0.89**	0.09	0.84**	0.88**	0.67**																			
I3M	-0.11*	-0.15**	-0.15**	-0.07	-0.11*	-0.09																		
4MOI3M	-0.13*	-0.06	-0.16**	-0.13*	-0.11*	-0.13*	0.44**																	
1MOI3M	0.21**	-0.03	0.20**	0.19**	0.17**	0.25**	0.15**	0.28**																
Leaf-GSL	0.95**	0.20**	0.87**	0.86**	0.80**	0.93**	0.03	-0.05	0.28**															
Seed-GSL	0.81**	0.25**	0.71**	0.73**	0.69**	0.75**	-0.19**	-0.23**	0.18**	0.79**														
TALI	0.96**	0.21**	0.90**	0.87**	0.81**	0.95**	-0.10*	-0.13*	0.25**	0.99**	0.82**													
TIND	-0.09	-0.14**	-0.13*	-0.06	-0.10	-0.07	0.98**	0.56**	0.24**	0.05	-0.18**	-0.08												
4C	0.96**	0.27**	0.93**	0.81**	0.80**	0.90**	-0.12*	-0.13*	0.23**	0.97**	0.81**	0.98**	-0.1											
5C	0.93**	0.15**	0.85**	0.9**	0.79**	0.97**	-0.09	-0.12*	0.26**	0.97**	0.8**	0.98**	-0.07	0.94**										
MSO	0.72**	0.64**	0.70**	0.58**	0.99	0.62**	-0.12*	-0.10*	0.16**	0.76**	0.67**	0.78**	-0.1	0.78**	0.75**									
OHAlk	0.99**	0.18**	0.81**	0.92**	0.75**	0.89**	-0.10	-0.13*	0.20**	0.95**	0.81**	0.96**	-0.09	0.95**	0.94**	0.71**								
Alkenyl	0.91**	0.09	0.93**	0.83**	0.71**	0.98**	-0.10	-0.14**	0.25**	0.95**	0.77**	0.97**	-0.08	0.95**	0.97**	0.67**	0.90**							
4MSO/4C	-0.4**	0.70**	-0.36**	-0.5**	-0.02	-0.57**	-0.05	0.02	-0.11*	-0.42**	-0.26**	-0.41**	-0.05	-0.37**	-0.45**	0.06	-0.42**	-0.51**						
5MSO/5C	-0.51**	0.50**	-0.46**	-0.64**	-0.08	-0.69**	0.02	0.10	-0.08	-0.52**	-0.37**	-0.53**	0.02	-0.48**	-0.58**	-0.02	-0.53**	-0.63**	0.83**					
4C/TALI	0.09	0.38**	0.22**	-0.20**	0.08	-0.12*	-0.09	-0.08	-0.02	0.03	0.04	0.03	-0.09	0.16**	-0.09	0.12*	0.04	-0.01	0.26**	0.27**				
OHAlk/TALI	0.63**	-0.01	0.28**	0.64**	0.32**	0.40**	-0.03	-0.07	0.07	0.49**	0.49**	0.48**	-0.03	0.50**	0.45**	0.29**	0.65**	0.38**	-0.31**	-0.31**	0.14**			
Alkenyl/TALI	0.23**	-0.37**	0.48**	0.25**	0.07	0.50**	-0.07	-0.09	0.04	0.31**	0.15**	0.34**	-0.07	0.30**	0.37**	0.02	0.23**	0.50**	-0.59**	-0.71**	-0.18**	-0.23**		
4MO/TIND	-0.08	0.04	-0.04	-0.09	-0.06	-0.08	-0.63**	0.28**	-0.01	-0.16**	-0.08	-0.08	-0.55**	-0.07	-0.08	-0.05	-0.08	-0.08	0.08	0.06	0.02	-0.09	0.03	
1MO/TIND	0.26**	0.06	0.28**	0.22**	0.23**	0.29**	-0.51**	-0.11*	0.74**	0.23**	0.30**	0.30**	-0.42**	0.28**	0.30**	0.22**	0.26**	0.30**	-0.07	-0.10	0.05	0.07	0.10	0.36**

*P<0.05;**P<0.01

Table S3 Analysis of variance (ANOVA) results of the 25 glucosinolate (GSL) traits among the association panel.

Trait	ANOVA			
	Genotype (G)	Environment (E)	G × E	Error
Leaf-GLS	2406.03**	410.26**	356.85**	247.83
Seed-GLS	2694664.43**	11945.78**	53122.15**	29,743.42
4OHB	208.56**	40.91**	52.33**	23.37
4MSO	2.52**	0.12**	0.53**	0.20
4BTEY	69.69**	10.18**	11.68**	11.68
5OHP	17.29**	0.12**	1.20**	1.06
5MSO	48.56**	7.61**	11.28**	5.65
5PTEY	449.88**	21.71**	51.09**	40.07
I3M	27.81**	0.67**	9.95*	15.32
4MOI3M	0.32**	0.17**	0.15**	0.12
1MOI3M	-	-	-	-
TALI	2313.78**	312.91**	334.44**	213.04
TIND	32.13**	3.43**	11.37*	18.15
4C	442.82**	98.52**	100.79**	53.47
5C	822.98**	60.27**	90.05**	64.92
MSO	58.34**	9.60**	12.94**	6.88
OHAlk	317.61**	45.45**	61.02**	29.90
Alkenyl	761.61**	61.61**	94.38**	77.91
4MSO/4C	34.36**	0.48**	5.19**	4.92
5MSO/5C	82.85**	1.40**	9.62**	10.64
4C/TALI	15.11**	5.91**	5.28**	5.50
OHAlk/TALI	20.18**	1.34**	4.40**	5.12
Alkenyl/TALI	40.81**	1.94**	7.22**	8.17
4MO/TIND	4.14**	0.07**	1.85**	1.95
1MO/TIND	-	-	-	-

*P < 0.01; **P < 0.0001.

Table S4 Summary of significant genome-wide association signals for the 25 glucosinolate (GSL) traits.

ID	Class	Trait	SNP	Chr.	Position	Major Allele	Minor Allele	MAF (521 lines)	MAF (257 lines)	$-\log_{10}$ (P)	PVE (%)[†]	Candidate gene
1	Total	Leaf-GSL	Bn-A03-p21329715	A3	20,095,857	A	G	0.24	0.33	5.91	8.23	*BnaA03g40190D*
2	Total	Leaf-GSL	Bn-A08-p12559372	A8	10,338,218	C	A	0.16	0.04	4.57	6.37	
3	Total	Leaf-GSL	Bn-A01-p9004629	A9	2,580,835	C	T	0.18	0.00	14.28	20.40	*BnaA09MYB28*[‡]
4	Total	Leaf-GSL	Bn-A10-p10454385	A10	11,834,653	A	C	0.05	0.02	4.67	6.11	
5	Total	Leaf-GSL	Bn-scaff_23432_1-p217818	C4	19,102,451	C	T	0.28	0.30	4.50	7.41	
6	Total	Leaf-GSL	Bn-scaff_15705_1-p2274493	C7	35,279,702	A	G	0.18	0.01	6.52	8.84	*BnaC07MYB28 (BnC07g0816690.1)*[§]
7	Total	Leaf-GSL	Bn-scaff_19783_1-p379086	C9	2,850,069	C	A	0.08	0.01	7.45	11.39	*BnaC09g05300D*
8	Total	Leaf-GSL	Bn-scaff_17799_1-p3050608	C9	39,518,182	A	G	0.08	0.08	5.73	7.91	
9	Total	Seed-GSL (366lines)	Bn-A08-p12794893	A8	10,478,871	C	T	0.19	0.05	7.55	10.73	
10	Total	Seed-GSL (366lines)	Bn-A09-p2733282	A9	2,677,575	A	G	0.22	0.01	25.36	42.60	*BnaA09MYB28*[‡]
11	Total	Seed-GSL (366lines)	Bn-scaff_17177_1-p441984	C2	44,768,013	G	T	0.21	0.03	10.19	15.09	*BnaC02MYB28*[‡]
12	Total	Seed-GSL (366lines)	Bn-scaff_15794_3-p75392	C3	55,706,974	G	A	0.15	0.02	4.79	6.23	
13	Total	Seed-GSL (366lines)	Bn-scaff_18181_1-p1849246	C7	34,322,798	A	G	0.19	0.05	6.61	9.37	*BnaC07MYB28 (BnC07g0816690.1)*[§]
14	Total	Seed-GSL (366lines)	Bn-A08-p8426380	C8	11,962,388	G	A	0.29	0.12	5.12	7.38	
15	Total	Seed-GSL (366lines)	Bn-scaff_19783_1-p379086	C9	2,850,069	C	A	0.08	0.01	11.20	17.68	*BnaC09g05300D*

ID	Class	Trait	SNP	Chr.	Position	Major Allele	Minor Allele	MAF (521 lines)	MAF (257 lines)	$-\log_{10}$ (P)	PVE (%)†	Candidate gene
16	Total	Seed-GSL (366lines)	Bn-scaff_22835_1-p619832	C9	11,113,843	C	T	0.28	0.38	4.77	6.52	*BnaC09g14380D*
17	Total	Seed-GSL (521lines)	Bn-A01-p970103	A1	588,064	C	T	0.15	0.11	4.53	4.75	
18	Total	Seed-GSL (521lines)	Bn-A09-p10577283	A2	24,468,610	C	T	0.15	0.07	4.73	5.25	*BnaA02g33530D*
19	Total	Seed-GSL (521lines)	Bn-A03-p21669774	A3	20,452,811	G	A	0.22	0.20	4.84	4.76	*BnaA03g40190D*
20	Total	Seed-GSL (521lines)	Bn-A02-p745468	A7	13,377,023	T	C	0.05	0.02	5.89	5.46	
21	Total	Seed-GSL (521lines)	Bn-A08-p12820786	A8	10,587,676	A	C	0.16	0.03	11.89	11.44	
22	Total	Seed-GSL (521lines)	Bn-A09-p2733282	A9	2,677,575	A	G	0.22	0.01	36.27	44.05	*BnaA09MYB28*‡
23	Total	Seed-GSL (521lines)	Bn-A10-p6896063	A10	8,474,298	C	T	0.10	0.06	4.55	4.33	
24	Total	Seed-GSL (521lines)	Bn-scaff_22749_1-p67780	C2	26,260,892	G	A	0.26	0.22	5.12	5.09	*BnaC02g27590D*
25	Total	Seed-GSL (521lines)	Bn-scaff_17177_1-p441984	C2	44,768,013	G	T	0.21	0.03	12.81	13.17	*BnaC02MYB28*‡
26	Total	Seed-GSL (521lines)	Bn-scaff_15794_3-p165426	C3	55,436,899	C	T	0.15	0.02	6.46	6.05	
27	Total	Seed-GSL (521lines)	Bn-scaff_16217_1-p181427	C4	22,294,107	A	G	0.42	0.48	4.73	4.52	
28	Total	Seed-GSL (521lines)	Bn-scaff_18181_1-p1849246	C7	34,322,798	A	G	0.19	0.05	9.29	9.25	*BnaC07MYB28 (BnC07g0816690.1)*§
29	Total	Seed-GSL (521lines)	Bn-scaff_19783_1-p379086	C9	2,850,069	C	A	0.08	0.01	11.33	12.13	*BnaC09g05300D*

ID	Class	Trait	SNP	Chr.	Position	Major Allele	Minor Allele	MAF (521 lines)	MAF (257 lines)	$-\log_{10}$ (P)	PVE (%)[†]	Candidate gene
30	Total	Seed-GSL (521lines)	Bn-scaff_22835_1-p619832	C9	11,113,843	C	T	0.28	0.38	7.30	7.03	*BnaC09g14380D*
31	Aliphatic	4OHB	Bn-A03-p21329715	A3	20,095,857	A	G	0.24	0.33	5.81	8.22	*BnaA03g40190D*
32	Aliphatic	4OHB	Bn-A04-p6264043	A4	7,476,656	C	T	0.20	0.14	4.54	6.75	
33	Aliphatic	4OHB	Bn-A08-p12814556	A8	10,579,999	C	T	0.17	0.02	6.72	9.65	
34	Aliphatic	4OHB	Bn-A01-p9004629	A9	2,580,835	C	T	0.18	0.00	14.56	20.90	*BnaA09MYB28*[‡]
35	Aliphatic	4OHB	Bn-A10-p10454385	A10	11,834,653	A	C	0.05	0.02	6.01	7.95	
36	Aliphatic	4OHB	Bn-scaff_17177_1-p441984	C2	44,768,013	G	T	0.21	0.03	4.61	6.82	*BnaC02MYB28*[‡]
37	Aliphatic	4OHB	Bn-scaff_15705_1-p2274493	C7	35,279,702	A	G	0.18	0.01	8.26	11.33	*BnaC07MYB28 (BnC07g0816690.1)*[§]
38	Aliphatic	4OHB	Bn-scaff_19783_1-p379086	C9	2,850,069	C	A	0.08	0.01	7.21	11.84	*BnaC09g05300D*
39	Aliphatic	4MSO	Bn-A02-p10515029	A2	7,376,035	C	T	0.33	0.40	4.66	6.27	
40	Aliphatic	4MSO	Bn-A02-p27149888	A2	24,469,045	A	G	0.10	0.10	4.70	6.34	
41	Aliphatic	4MSO	Bn-A03-p26740723	A3	25,162,686	A	C	0.25	0.25	6.67	8.94	
42	Aliphatic	4MSO	Bn-A03-p29956906	A3	27,989,280	A	G	0.10	0.09	4.88	6.43	
43	Aliphatic	4MSO	Bn-A05-p5627413	A5	5,376,033	C	T	0.06	0.07	7.78	10.40	
44	Aliphatic	4MSO	Bn-A05-p7187425	A5	6,615,357	G	A	0.10	0.12	4.94	6.65	*BnaA05g11170D*
45	Aliphatic	4MSO	Bn-Scaffold000172-p99636	A5	10,429,028	C	T	0.08	0.08	6.19	8.96	
46	Aliphatic	4MSO	Bn-A06-p21933870	A6	20,956,440	G	T	0.16	0.14	6.58	9.06	*BnaA06g31890D*
47	Aliphatic	4MSO	Bn-A08-p13214532	A8	10,959,920	G	A	0.05	0.01	7.04	9.34	
48	Aliphatic	4MSO	Bn-A08-p19191695	A8	16,530,607	T	G	0.10	0.12	13.00	20.43	*BnaA08g24880D*
49	Aliphatic	4MSO	Bn-A10-p1611973	A10	2,093,174	T	C	0.06	0.02	4.95	6.92	
50	Aliphatic	4MSO	Bn-scaff_17515_1-p195141	C1	34,710,942	G	T	0.12	0.12	4.51	6.46	

ID	Class	Trait	SNP	Chr.	Position	Major Allele	Minor Allele	MAF (521 lines)	MAF (257 lines)	$-\log_{10}$ (P)	PVE (%)†	Candidate gene
51	Aliphatic	4MSO	Bn-scaff_16369_1-p181890	C2	18,594,801	T	G	0.17	0.18	4.53	6.43	
52	Aliphatic	4MSO	Bn-scaff_16002_1-p1806874	C3	12,569,994	A	C	0.07	0.05	6.98	9.24	
53	Aliphatic	4MSO	Bn-scaff_16002_1-p772984	C3	13,554,815	G	A	0.05	0.07	6.81	10.25	
54	Aliphatic	4MSO	Bn-scaff_18206_1-p514412	C3	53,064,565	G	A	0.17	0.07	5.04	7.06	
55	Aliphatic	4MSO	Bn-scaff_17592_1-p888125	C4	13,045,030	A	G	0.05	0.05	4.40	5.94	
56	Aliphatic	4MSO	Bn-scaff_19253_1-p344350	C4	15,342,125	G	T	0.17	0.18	6.99	9.31	
57	Aliphatic	4MSO	Bn-scaff_16394_1-p79652	C4	31,478,981	C	T	0.13	0.11	4.96	6.69	*BnaC04g29320D*
58	Aliphatic	4MSO	Bn-scaff_20270_1-p1210276	C5	41,643,254	G	A	0.08	0.09	14.40	20.74	
59	Aliphatic	4BTEY	Bn-A01-p9866468	A1	8,491,979	G	A	0.04	0.01	5.34	6.98	
60	Aliphatic	4BTEY	Bn-A03-p21329715	A3	20,095,857	A	G	0.24	0.33	5.90	7.91	*BnaA03g40190D*
61	Aliphatic	4BTEY	Bn-A04-p12236240	A4	13,224,285	C	A	0.38	0.38	4.92	6.62	
62	Aliphatic	4BTEY	Bn-A04-p18089854	A4	18,176,833	C	T	0.25	0.17	4.54	6.42	*BnaA04g24160D*
63	Aliphatic	4BTEY	Bn-A05-p2067722	A5	2,199,180	G	A	0.22	0.19	4.63	6.23	
64	Aliphatic	4BTEY	Bn-A07-p11324360	A7	12,688,030	T	C	0.06	0.06	4.58	6.00	
65	Aliphatic	4BTEY	Bn-A08-p13239621	A8	10,991,702	T	C	0.21	0.10	4.80	6.41	
66	Aliphatic	4BTEY	Bn-A08-p19676653	A8	17,007,109	T	C	0.08	0.04	4.90	7.88	*BnaA08g24880D*
67	Aliphatic	4BTEY	Bn-A09-p3994064	A9	3,999,691	T	C	0.14	0.02	8.12	11.17	*BnaA09MYB28*‡
68	Aliphatic	4BTEY	Bn-scaff_19183_1-p11952	C1	21,296,423	G	T	0.16	0.19	5.53	7.47	
69	Aliphatic	4BTEY	Bn-scaff_21820_1-p577537	C1	25,080,630	C	T	0.15	0.18	4.73	6.47	
70	Aliphatic	4BTEY	Bn-scaff_22115_1-p155376	C1	35,743,833	A	G	0.04	0.03	4.46	7.43	
71	Aliphatic	4BTEY	Bn-scaff_16352_1-p25266	C3	15,130,104	A	G	0.17	0.18	4.49	5.84	

ID	Class	Trait	SNP	Chr.	Position	Major Allele	Minor Allele	MAF (521 lines)	MAF (257 lines)	$-\log_{10}$ (P)	PVE (%)†	Candidate gene
72	Aliphatic	4BTEY	Bn-scaff_19253_1-p24802	C4	15,008,701	C	T	0.22	0.19	4.54	5.98	
73	Aliphatic	4BTEY	Bn-scaff_16217_1-p471484	C4	22,044,497	T	G	0.07	0.01	5.24	7.03	
74	Aliphatic	4BTEY	Bn-scaff_20901_1-p583439	C5	3,468,004	T	G	0.07	0.04	4.65	6.00	
75	Aliphatic	4BTEY	Bn-scaff_18807_1-p678223	C6	30,163,619	T	C	0.49	0.49	4.58	7.42	
76	Aliphatic	4BTEY	Bn-scaff_19724_1-p166186	C7	26,237,235	C	A	0.06	0.05	4.52	5.86	
77	Aliphatic	4BTEY	Bn-scaff_15705_1-p2274493	C7	35,279,702	A	G	0.18	0.01	7.96	10.64	*BnaC07MYB28 (BnC07g0816690.1)*§
78	Aliphatic	4BTEY	Bn-scaff_16110_1-p1936428	C7	42,951,950	T	C	0.13	0.12	4.41	5.82	
79	Aliphatic	4BTEY	Bn-scaff_16197_1-p2366422	C8	31,829,862	C	T	0.08	0.07	4.42	5.70	
80	Aliphatic	4BTEY	Bn-scaff_15650_1-p908638	C9	17,100,181	A	C	0.05	0.03	4.88	6.39	
81	Aliphatic	5OHP	Bn-A03-p14176469	A3	13,373,806	C	T	0.14	0.10	5.07	6.96	*BnaA03g25870D*
82	Aliphatic	5OHP	Bn-A03-p17986611	A3	16,993,754	A	G	0.05	0.02	4.53	6.27	*BnaA03g35400D*
83	Aliphatic	5OHP	Bn-A03-p22028778	A3	20,848,772	A	C	0.11	0.10	8.55	12.33	*BnaA03g40190D*
84	Aliphatic	5OHP	Bn-A07-p14820942	A7	16,693,589	A	G	0.26	0.22	4.46	6.22	*BnaA07g21440D*
85	Aliphatic	5OHP	Bn-A08-p16171460	A8	13,632,493	C	T	0.46	0.44	5.88	8.21	*BnaA08g16110D*
86	Aliphatic	5OHP	Bn-A01-p9004629	A9	2,580,835	C	T	0.18	0.00	12.33	17.30	*BnaA09MYB28*‡
87	Aliphatic	5OHP	Bn-scaff_21884_1-p650617	C1	37,464,011	C	T	0.05	0.00	7.77	11.38	*BnaC01g39030D*
88	Aliphatic	5OHP	Bn-scaff_16002_1-p2153239	C3	12,243,471	G	A	0.09	0.12	6.00	9.39	
89	Aliphatic	5OHP	Bn-scaff_16095_1-p43615	C4	9,993,476	G	A	0.06	0.03	5.06	6.69	*BnaC04g12860D*
90	Aliphatic	5OHP	Bn-scaff_15892_1-p365584	C6	26,260,466	C	A	0.13	0.10	8.79	12.06	*BnaC06g24240D*

ID	Class	Trait	SNP	Chr.	Position	Major Allele	Minor Allele	MAF (521 lines)	MAF (257 lines)	$-\log_{10}$ (P)	PVE (%)†	Candidate gene
91	Aliphatic	5OHP	Bn-scaff_15705_1-p2279820	C7	35,285,037	C	T	0.17	0.01	4.39	5.85	*BnaC07MYB28 (BnC07g0816690.1)*§
92	Aliphatic	5OHP	Bn-scaff_17190_1-p9206	C9	3,592,310	A	G	0.18	0.06	7.61	11.45	*BnaC09g05300D*
93	Aliphatic	5OHP	Bn-scaff_17367_1-p737183	C9	32,670,669	G	A	0.19	0.22	4.71	6.53	
94	Aliphatic	5MSO	Bn-A03-p21941473	A3	20,740,672	T	C	0.06	0.02	5.79	7.66	*BnaA03g40190D*
95	Aliphatic	5MSO	Bn-A08-p12814556	A8	10,579,999	C	T	0.17	0.02	7.22	10.31	
96	Aliphatic	5MSO	Bn-A09-p2733282	A9	2,677,575	A	G	0.22	0.01	9.17	12.90	*BnaA09MYB28*‡
97	Aliphatic	5MSO	Bn-scaff_17799_1-p853567	C6	35,737,877	A	G	0.10	0.04	6.85	9.21	*BnaC06g38820D; BnaC06g38830D; BnaC06g38840D; BnaC06g38850D*
98	Aliphatic	5MSO	Bn-scaff_18181_1-p1849246	C7	34,322,798	A	G	0.19	0.05	5.02	6.89	*BnaC07MYB28 (BnC07g0816690.1)*§
99	Aliphatic	5MSO	Bn-scaff_19783_1-p379086	C9	2,850,069	C	A	0.08	0.01	5.90	9.11	*BnaC09g05300D*
100	Aliphatic	5PTEY	Bn-A03-p21329715	A3	20,095,857	A	G	0.24	0.33	5.07	6.85	*BnaA03g40190D*
101	Aliphatic	5PTEY	Bn-A04-p10630934	A4	11,775,918	G	A	0.07	0.04	4.66	6.33	
102	Aliphatic	5PTEY	Bn-A01-p9004629	A9	2,580,835	C	T	0.18	0.00	11.25	15.81	*BnaA09MYB28*‡
103	Aliphatic	5PTEY	Bn-A10-p10454385	A10	11,834,653	A	C	0.05	0.02	4.44	5.83	
104	Aliphatic	5PTEY	Bn-scaff_21884_1-p650617	C1	37,464,011	C	T	0.05	0.00	4.51	6.28	*BnaC01g39030D*
105	Aliphatic	5PTEY	Bn-scaff_16352_1-p25266	C3	15,130,104	A	G	0.17	0.18	4.40	5.85	
106	Aliphatic	5PTEY	Bn-scaff_23432_1-p217818	C4	19,102,451	C	T	0.28	0.30	5.30	8.50	
107	Aliphatic	5PTEY	Bn-scaff_19724_1-p167646	C7	26,235,776	G	A	0.06	0.05	4.58	6.00	
108	Aliphatic	5PTEY	Bn-scaff_15705_1-p2317575	C7	35,328,395	A	C	0.27	0.14	7.15	10.12	*BnaC07MYB28 (BnC07g0816690.1)*§

ID	Class	Trait	SNP	Chr.	Position	Major Allele	Minor Allele	MAF (521 lines)	MAF (257 lines)	$-log_{10}$ (P)	PVE (%)[†]	Candidate gene
109	Aliphatic	5PTEY	Bn-scaff_16197_1-p2366422	C8	31,829,862	C	T	0.08	0.07	4.41	5.87	
110	Aliphatic	5PTEY	Bn-scaff_17190_1-p9206	C9	3,592,310	A	G	0.18	0.06	7.42	11.08	*BnaC09g05300D*
111	Aliphatic	5PTEY	Bn-scaff_17799_1-p3050608	C9	39,518,182	A	G	0.08	0.08	8.22	11.54	
112	Indolic	I3M	Bn-A02-p25079431	A2	23,154,160	A	C	0.30	0.29	4.52	6.40	*BnaA02MYB34 (BnA02g0086460.1)*[§]
113	Indolic	I3M	Bn-scaff_17177_1-p532521	C2	44,698,711	A	G	0.26	0.05	5.00	6.99	*BnaC02g41860D*
114	Indolic	1MOI3M	Bn-scaff_16394_2-p553789	C3	50,754,150	T	C	0.47	0.38	6.55	9.30	*BnaC03g61420D*
115	Aliphatic	TALI	Bn-A03-p21329715	A3	20,095,857	A	G	0.24	0.33	6.51	9.04	*BnaA03g40190D*
116	Aliphatic	TALI	Bn-A08-p12559372	A8	10,338,218	C	A	0.16	0.04	4.60	6.43	
117	Aliphatic	TALI	Bn-A01-p9004629	A9	2,580,835	C	T	0.18	0.00	13.90	19.84	*BnaA09MYB28*[‡]
118	Aliphatic	TALI	Bn-A10-p10454385	A10	11,834,653	A	C	0.05	0.02	4.64	6.07	
119	Aliphatic	TALI	Bn-scaff_23432_1-p217818	C4	19,102,451	C	T	0.28	0.30	4.54	7.54	
120	Aliphatic	TALI	Bn-scaff_15705_1-p2274493	C7	35,279,702	A	G	0.18	0.01	6.72	9.14	*BnaC07MYB28 (BnC07g0816690.1)*[§]
121	Aliphatic	TALI	Bn-scaff_19783_1-p379086	C9	2,850,069	C	A	0.08	0.01	7.67	11.69	*BnaC09g05300D*
122	Aliphatic	TALI	Bn-scaff_17799_1-p3050608	C9	39,518,182	A	G	0.08	0.08	6.10	8.43	
123	Indolic	TIND	Bn-A02-p25079431	A2	23,154,160	A	C	0.30	0.29	4.60	6.47	*BnaA02MYB34 (BnA02g0086460.1)*[§]
124	Indolic	TIND	Bn-scaff_17177_1-p532521	C2	44,698,711	A	G	0.26	0.05	5.23	7.34	*BnaC02g41860D*
125	Aliphatic	4C	Bn-A03-p21329715	A3	20,095,857	A	G	0.24	0.33	7.28	10.21	*BnaA03g40190D*
126	Aliphatic	4C	Bn-A08-p12814556	A8	10,579,999	C	T	0.17	0.02	5.32	7.60	
127	Aliphatic	4C	Bn-A09-p2733282	A9	2,677,575	A	G	0.22	0.01	13.43	19.26	*BnaA09MYB28*[‡]

ID	Class	Trait	SNP	Chr.	Position	Major Allele	Minor Allele	MAF (521 lines)	MAF (257 lines)	$-log_{10}$ (P)	PVE (%)[†]	Candidate gene
128	Aliphatic	4C	Bn-A10-p10454385	A10	11,834,653	A	C	0.05	0.02	5.21	6.83	
129	Aliphatic	4C	Bn-A10-p13796417	A10	13,831,831	C	T	0.17	0.15	5.14	7.22	
130	Aliphatic	4C	Bn-scaff_15705_1-p2274493	C7	35,279,702	A	G	0.18	0.01	8.91	12.27	*BnaC07MYB28 (BnC07g0816690.1)*[§]
131	Aliphatic	4C	Bn-scaff_19783_1-p379086	C9	2,850,069	C	A	0.08	0.01	7.50	12.62	*BnaC09g05300D*
132	Aliphatic	5C	Bn-A03-p21329715	A3	20,095,857	A	G	0.24	0.33	5.05	6.86	*BnaA03g40190D*
133	Aliphatic	5C	Bn-A01-p9004629	A9	2,580,835	C	T	0.18	0.00	13.85	19.64	*BnaA09MYB28*[‡]
134	Aliphatic	5C	Bn-scaff_21884_1-p650617	C1	37,464,011	C	T	0.05	0.00	5.17	7.20	*BnaC01g39030D*
135	Aliphatic	5C	Bn-scaff_17177_1-p381225	C2	44,843,894	T	A	0.25	0.04	4.38	6.33	*BnaC02MYB28*[‡]
136	Aliphatic	5C	Bn-scaff_23432_1-p217818	C4	19,102,451	C	T	0.28	0.30	5.19	8.54	
137	Aliphatic	5C	Bn-scaff_15705_1-p2317575	C7	35,328,395	A	C	0.27	0.14	5.95	8.23	*BnaC07MYB28 (BnC07g0816690.1)*[§]
138	Aliphatic	5C	Bn-scaff_17190_1-p9206	C9	3,592,310	A	G	0.18	0.06	7.47	11.11	*BnaC09g05300D*
139	Aliphatic	5C	Bn-scaff_17799_1-p3050608	C9	39,518,182	A	G	0.08	0.08	7.53	10.69	
140	Aliphatic	MSO	Bn-A03-p21941473	A3	20,740,672	T	C	0.06	0.02	5.33	7.08	*BnaA03g40190D*
141	Aliphatic	MSO	Bn-A06-p1658095	A6	1,756,502	G	T	0.12	0.11	4.70	6.80	
142	Aliphatic	MSO	Bn-A08-p12814556	A8	10,579,999	C	T	0.17	0.02	7.04	10.21	
143	Aliphatic	MSO	Bn-A09-p3097420	A9	3,031,274	T	G	0.24	0.02	8.11	11.39	*BnaA09MYB28*[‡]
144	Aliphatic	MSO	Bn-scaff_17799_1-p853567	C6	35,737,877	A	G	0.10	0.04	5.74	7.66	*BnaC06g38820D; BnaC06g38830D; BnaC06g38840D; BnaC06g38850D*
145	Aliphatic	MSO	Bn-scaff_18181_1-p1849246	C7	34,322,798	A	G	0.19	0.05	4.44	6.15	*BnaC07MYB28 (BnC07g0816690.1)*[§]

ID	Class	Trait	SNP	Chr.	Position	Major Allele	Minor Allele	MAF (521 lines)	MAF (257 lines)	-$\log_{10}$ (P)	PVE (%)[†]	Candidate gene
146	Aliphatic	MSO	Bn-scaff_19783_1-p379086	C9	2,850,069	C	A	0.08	0.01	5.10	8.20	*BnaC09g05300D*
147	Aliphatic	OHAlk	Bn-A03-p21329715	A3	20,095,857	A	G	0.24	0.33	5.24	7.38	*BnaA03g40190D*
148	Aliphatic	OHAlk	Bn-A08-p12814556	A8	10,579,999	C	T	0.17	0.02	6.27	8.87	
149	Aliphatic	OHAlk	Bn-A01-p9004629	A9	2,580,835	C	T	0.18	0.00	14.96	21.45	*BnaA09MYB28*[‡]
150	Aliphatic	OHAlk	Bn-A10-p5673588	A10	5,318,035	T	C	0.33	0.29	4.39	6.09	
151	Aliphatic	OHAlk	Bn-A10-p10454385	A10	11,834,653	A	C	0.05	0.02	5.50	7.26	
152	Aliphatic	OHAlk	Bn-scaff_21884_1-p650617	C1	37,464,011	C	T	0.05	0.00	4.41	6.15	*BnaC01g39030D*
153	Aliphatic	OHAlk	Bn-scaff_17177_1-p441984	C2	44,768,013	G	T	0.21	0.03	4.80	7.15	*BnaC02MYB28*[‡]
154	Aliphatic	OHAlk	Bn-scaff_16002_1-p2153239	C3	12,243,471	G	A	0.09	0.12	4.44	6.45	
155	Aliphatic	OHAlk	Bn-scaff_19821_1-p294505	C4	12,685,781	T	C	0.08	0.06	4.81	6.46	
156	Aliphatic	OHAlk	Bn-scaff_16116_1-p605377	C6	28,471,888	A	G	0.16	0.11	4.50	6.12	
157	Aliphatic	OHAlk	Bn-scaff_15705_1-p2274493	C7	35,279,702	A	G	0.18	0.01	7.48	10.15	*BnaC07MYB28 (BnC07g0816690.1)*[§]
158	Aliphatic	OHAlk	Bn-scaff_19783_1-p379086	C9	2,850,069	C	A	0.08	0.01	6.91	11.07	*BnaC09g05300D*
159	Aliphatic	Alkenyl	Bn-A03-p6404903	A3	5,727,023	C	A	0.07	0.05	4.45	5.86	
160	Aliphatic	Alkenyl	Bn-A03-p21329715	A3	20,095,857	A	G	0.24	0.33	6.17	8.41	*BnaA03g40190D*
161	Aliphatic	Alkenyl	Bn-A04-p10630934	A4	11,775,918	G	A	0.07	0.04	4.71	6.32	
162	Aliphatic	Alkenyl	Bn-A08-p19676653	A8	17,007,109	T	C	0.08	0.04	4.93	8.50	*BnaA08g24880D*
163	Aliphatic	Alkenyl	Bn-A09-p2733282	A9	2,677,575	A	G	0.22	0.01	10.20	14.35	*BnaA09MYB28*[‡]
164	Aliphatic	Alkenyl	Bn-scaff_16352_1-p25266	C3	15,130,104	A	G	0.17	0.18	5.48	7.31	
165	Aliphatic	Alkenyl	Bn-scaff_23432_1-p217818	C4	19,102,451	C	T	0.28	0.30	4.95	7.79	

ID	Class	Trait	SNP	Chr.	Position	Major Allele	Minor Allele	MAF (521 lines)	MAF (257 lines)	$-\log_{10}$ (P)	PVE (%)[†]	Candidate gene
166	Aliphatic	Alkenyl	Bn-scaff_19724_1-p167646	C7	26,235,776	G	A	0.06	0.05	5.06	6.63	
167	Aliphatic	Alkenyl	Bn-scaff_15705_1-p2317575	C7	35,328,395	A	C	0.27	0.14	7.45	10.50	*BnaC07MYB28 (BnC07g0816690.1)*[§]
168	Aliphatic	Alkenyl	Bn-scaff_16197_1-p2366422	C8	31,829,862	C	T	0.08	0.07	5.50	7.36	
169	Aliphatic	Alkenyl	Bn-scaff_17190_1-p9206	C9	3,592,310	A	G	0.18	0.06	6.74	9.93	*BnaC09g05300D*
170	Aliphatic	Alkenyl	Bn-scaff_17339_1-p38035	C9	21,388,332	T	C	0.20	0.18	4.43	6.35	*BnaC09g23540D*
171	Aliphatic	Alkenyl	Bn-scaff_17799_1-p3050608	C9	39,518,182	A	G	0.08	0.08	7.73	10.63	
172	Aliphatic	4MSO/4C	Bn-A03-p13975945	A3	13,117,998	G	A	0.11	0.08	5.02	6.61	*BnaA03g25870D*
173	Aliphatic	4MSO/4C	Bn-A03-p21329715	A3	20,095,857	A	G	0.24	0.33	10.85	15.75	*BnaA03g39680D; BnaA03g39710D; BnaA03g39720D*
174	Aliphatic	4MSO/4C	Bn-scaff_22728_1-p305373	C3	6,208,380	T	C	0.25	0.26	4.91	6.55	
175	Aliphatic	4MSO/4C	Bn-scaff_18322_1-p1763220	C3	7,364,962	A	G	0.28	0.28	4.65	6.48	
176	Aliphatic	4MSO/4C	Bn-scaff_16002_1-p772984	C3	13,554,815	G	A	0.05	0.07	6.98	11.01	
177	Aliphatic	5MSO/5C	Bn-A03-p21329715	A3	20,095,857	A	G	0.24	0.33	16.69	25.95	*BnaA03g39680D; BnaA03g39710D; BnaA03g39720D*
178	Aliphatic	5MSO/5C	Bn-A03-p22014622	C7	36,133,823	T	G	0.31	0.32	4.54	6.42	
179	Aliphatic	4C/TALI	Bn-A03-p21787407	A3	20,570,287	G	A	0.24	0.29	7.54	10.98	*BnaA03g39680D; BnaA03g39710D; BnaA03g39720D*
180	Aliphatic	4C/TALI	Bn-scaff_16002_1-p734704	C3	13,588,011	T	C	0.10	0.08	4.69	6.96	

ID	Class	Trait	SNP	Chr.	Position	Major Allele	Minor Allele	MAF (521 lines)	MAF (257 lines)	$-log_{10}$ (P)	PVE (%)[†]	Candidate gene
181	Aliphatic	OHAlk/TALI	Bn-A03-p7688578	A3	6,987,172	C	T	0.24	0.24	9.48	14.05	*BnaA03g15200D; BnaA03g15210D*
182	Aliphatic	OHAlk/TALI	Bn-A09-p3029767	A9	2,949,845	A	G	0.23	0.02	4.54	5.90	*BnaA09MYB28*[‡]
183	Aliphatic	OHAlk/TALI	Bn-scaff_23821_1-p122129	C6	30,084,174	G	A	0.36	0.43	4.78	7.34	
184	Aliphatic	Alkenyl/TALI	Bn-A03-p7642692	A3	6,943,540	A	G	0.33	0.35	4.73	6.69	*BnaA03g15200D; BnaA03g15210D*
185	Aliphatic	Alkenyl/TALI	Bn-A03-p21329715	A3	20,095,857	A	G	0.24	0.33	16.77	25.42	*BnaA03g39680D; BnaA03g39710D; BnaA03g39720D*
186	Aliphatic	Alkenyl/TALI	Bn-A05-p1554943	A5	1,719,644	A	G	0.32	0.32	4.71	6.32	
187	Aliphatic	Alkenyl/TALI	Bn-A03-p21980336	C7	36,105,765	G	C	0.48	0.47	4.38	6.12	
188	Indolic	4MO/TIND	Bn-A07-p15428458	A7	17,342,826	T	C	0.46	0.48	5.05	7.01	*BnaA07g21250D*
189	Indolic	4MO/TIND	Bn-scaff_15892_1-p1298704	C6	27,150,846	C	T	0.06	0.03	4.84	7.71	*BnaC06g25040D; BnaC06g25050D*
190	Indolic	1MO/TIND	Bn-A04-p13711282	A4	14,351,459	A	C	0.14	0.09	4.69	6.03	
191	Indolic	1MO/TIND	Bn-scaff_16394_2-p510062	C3	50,830,989	C	T	0.44	0.37	5.74	7.39	*BnaC03g61420D*

[†]Percentage of phenotypic variance explained by lead SNP marker.

[‡]Candidate genes were deleted from low-glucosinolate accessions according to Harper *et al*., (2012).

[§]Candidate genes in bracket were based on the *B. napus* "ZS11" reference genome (Sun *et al*., 2017).

Table S5 Primers used in this study.

Primer	Sequence（5’ to 3’）	Application
BnA3.MYB28-1F	GAAGGCTAAAGAAGCATGTGGTG	*BnaA03g40190D* cloning
BnA3.MYB28-1R	CCTCAAAAACACCCAAGAATGC	
BnA3.MYB28-2F	AACATGAAAACACCTTGCAGCT	*BnaA03g40190D* cloning; Genotyping of InDel1356
BnA3.MYB28-2R	GCCAAATGACTTGCGCTTAAG	
InDel4F	AAAATATACAATAACTACAAGCTGGC	Genotyping of InDel4
InDel4R	AGAGGCGTAAATTGTTTGATGC	
InDel13F	TCAGATGCATCAGAGTTCTCATCA	Genotyping of InDel13
InDel13R	CCTCAAAAACACCCAAGAATGC	
RealACT2F	CTGGAATTGCTGACCGTATGAG	qRT-PCR for *BnACTIN*
RealACT2R	ATCTGTTGGAAAGTGCTGAGGG	
RealBnA3.MYB28F	AATGTCGAGTATGGTCATGATCTTC	qRT-PCR for *BnaA03g40190D*
RealBnA3.MYB28R	CCAACCCTCGAAATTTTCAATC	
